# Supplementary material for: Analysis of PPARγ Signaling Activity in Psoriasis
Source: Int J Mol Sci. 2021 Aug 10;22(16):8603. doi: 10.3390/ijms22168603 (PMC8395241; doi:10.3390/ijms22168603)
Supplement: Supplementary file 1 [file ijms-22-08603-s001.zip › Supplemental materials_Analysis of PPARg signaling activity in psoriasis/Pathway models/Models images and html files/Interleukin-17 and interleukin-22 signaling in psoriasis/11922.html]

VEGFA


# Protein VEGFA

|  |  |
| --- | --- |
| URN | urn:agi-llid:7422 |
| Total Entities | 0 |
| Connectivity | 18176 |
| Name | VEGFA |
| Description | vascular endothelial growth factor A |
| Notes | This gene is a member of the PDGF/VEGF growth factor family. It encodes a heparin-binding protein, which exists as a disulfide-linked homodimer. This growth factor induces proliferation and migration of vascular endothelial cells, and is essential for both physiological and pathological angiogenesis. Disruption of this gene in mice resulted in abnormal embryonic blood vessel formation. This gene is upregulated in many known tumors and its expression is correlated with tumor stage and progression. Elevated levels of this protein are found in patients with POEMS syndrome, also known as Crow-Fukase syndrome. Allelic variants of this gene have been associated with microvascular complications of diabetes 1 (MVCD1) and atherosclerosis. Alternatively spliced transcript variants encoding different isoforms have been described. There is also evidence for alternative translation initiation from upstream non-AUG (CUG) codons resulting in additional isoforms. A recent study showed that a C-terminally extended isoform is produced by use of an alternative in-frame translation termination codon via a stop codon readthrough mechanism, and that this isoform is antiangiogenic. Expression of some isoforms derived from the AUG start codon is regulated by a small upstream open reading frame, which is located within an internal ribosome entry site. [provided by RefSeq, Nov 2015] |
| Primary Cell Localization | Extracellular |
| Class | Ligand |
| ObjectType | Protein |

---

|  |  |
| --- | --- |
| Pathway | Osteoarthritis |
|  | Somatostatin Receptors Signaling |
|  | Eosinophil Survival by Cytokine Signaling |
|  | Eosinophil Activation |
|  | Mast-Cell Activation via IgE Signaling |
|  | Mast-Cell Activation without Degranulation through CRHR1 Signaling |
|  | Mast-Cell Activation without Degranulation |
|  | anti-Inflammatory Function of Macrophage M2 Lineage |
|  | VEGFA/NOTCH1/WNT Cross-talk in Blood Vessel Sprouting and Branching in Cancer |
|  | Vascular Endothelial Cell Activation by NO |
|  | Vascular Smooth Muscle Cell/Pericyte Migration and Proliferation |
|  | Vascular Endothelial Cell Activation by Blood Coagulation Factors |
|  | Vascular Endothelial Cell Activation by Growth Factors |
|  | Vascular Endothelial Cell Permeability Activation |
|  | Nociception Expression Targets Signaling |
|  | CCR1 Expression Targets |
|  | IL1B Expression Targets -> Nociception |
|  | Hemapoietic Stem Cell -> Lymphoid Multipotent Progenitor Surface Expression Markers |
|  | Proteins Involved in Atherosclerosis |
|  | Proteins Involved in Dilated Cardiomyopathy |
|  | Proteins Involved in Arterial Hypertension |
|  | Arterial Hypertension |
|  | Proteins Involved in Pulmonary Hypertension |
|  | Proteins Involved in Hypoxia-Induced Pulmonary Hypertension |
|  | Endothelial Cell Dysfunction in Pulmonary Hypertension |
|  | BMP2 and Canonical WNT Signaling in Pulmonary Artery Endothelial Cells |
|  | Proteins Involved in Myocardial Ischemia |
|  | Proteins Involved in Myocarditis |
|  | Proteins Involved in Androgenetic Alopecia |
|  | Proteins with Altered Expression in Melanoma |
|  | Melanoma |
|  | MITF as a Regulator of Melanoma Cell Development |
|  | Proteins Involved in Melanoma |
|  | IL17 Signaling in Psoriasis |
|  | Proteins Involved in Psoriasis |
|  | Genes with Mutations Associated with Psoriasis |
|  | Dendritic Cells Function in Psoriasis |
|  | Proteins Involved in Diabetes Mellitus Type 2 |
|  | Peripheral Tissue Microangiopathy |
|  | Endothelial Cell Dysfunction in Progressive Diabetic Nephropathy |
|  | Proteins Involved in Diabetic Nephropathy |
|  | Proteins Involved in Diabetic Neuropathy |
|  | Microangiopathy in Diabetic Neuropathy |
|  | Proliferative Diabetic Retinopathy |
|  | Proteins Involved in non-Alcoholic Fatty Liver Disease |
|  | Proteins Involved in Hashimoto's Thyroiditis |
|  | Proteins Involved in Obesity |
|  | Proteins Involved in Prolactinoma |
|  | Prolactinoma Overiew |
|  | Proteins Involved in Colorectal Neoplasms |
|  | Cetuximab Resistance in Colorectal Cancer |
|  | Metastatic Colorectal Cancer |
|  | Proteins Involved in Inflammatory Bowel Diseases |
|  | CagA Phosphorylation Dependent Signaling |
|  | Proteins Involved in Ulcerative Colitis |
|  | Proteins Involved in B-Cell Chronic Lymphocytic Leukemia |
|  | Proteins Involved in Hodgkin Lymphoma |
|  | TNFR/NF-kB Classical Pathway in Multiple Myeloma Plasma Cells |
|  | Osteoclast Activation in Multiple Myeloma |
|  | IL6/IGF1/VEGFA Signaling in Multiple Myeloma |
|  | Multiple Myeloma |
|  | Proteins Involved in Multiple Myeloma |
|  | NOTCH Signaling in Multiple Myeloma |
|  | Proteins Involved in Thrombophilia |
|  | Vascularization in Hepatocellular Carcinoma |
|  | Proteins Involved in Hepatocellular Carcinoma |
|  | Hepatocellular Carcinoma |
|  | Growth Factor Signaling in Hepatocellular Carcinoma |
|  | Proteins Involved in Pancreatic Cancer |
|  | Pancreatic Ductal Carcinoma |
|  | Growth Factor Signaling in Pancreatic Neoplasms |
|  | ADAM33 Role in Asthma |
|  | Eosinophil Survival in Asthma |
|  | Mast-Cell Activation in Asthma |
|  | CFTR Facilitates Oxidative Stress in Airway Epithelium in CF |
|  | Proteins Involved in Osteoarthritis |
|  | Proteins Involved in Polycystic Ovary Syndrome |
|  | Synovial Fibroblast Activation in Psoriatic Arthritis |
|  | Keratinocyte Activation in Psoriatic Arthritis |
|  | Synovial Fibroblast Activation by Citokines in Rheumatoid Arthritis |
|  | TLR2 Induced Synovial Fibroblast Activation in Rheumatoid Arthritis |
|  | Proteins Involved in Rheumatoid Arthritis |
|  | Proteins Involved in Systemic Scleroderma |
|  | Skin Fibrosis |
|  | Estrogen Effects on Pregnancy Gingivitis |
|  | Proteins Involved in Periodontitis |
|  | Proteins with Altered Expression in Osteoarthritis |
|  | Proteins Involved in Osteoporosis |
|  | Proteins with Altered Expression in Psoriatic Arthritis |
|  | Proteins Involved in Epilepsy |
|  | Proteins Involved in Glioblastoma |
|  | Proteins Involved in Astrocytoma |
|  | Proteins Involved in Glioma |
|  | Growth Factor Signaling in Neuroblastoma |
|  | Neuroblastoma |
|  | ALK Associated Neuroblastoma |
|  | Proteins Involved in Neuroblastoma |
|  | SOD1 Mutation |
|  | Proteins with Altered Expression in Amyotrophic Lateral Sclerosis |
|  | Proteins Involved in Amyotrophic Lateral Sclerosis |
|  | Neuroinflammation in Amyotrophic Lateral Sclerosis |
|  | Proteins Involved in Multiple Sclerosis |
|  | Genes with Mutations Associated with Vascular Dementia |
|  | Proteins Involved in Vascular Dementia |
|  | Proteins Involved in Breast Cancer Related to WNT Signaling Pathway |
|  | Proteins Involved in Breast Cancer Related to NOTCH Signaling Pathway |
|  | WNT Signaling in Breast Cancer |
|  | Proteins Involved in Breast Cancer Related to ERBB2/VEGFR/Akt Signaling Pathway |
|  | ERBB/VEGFR/Akt Signaling in Breast Cancer |
|  | ESR1/ERBB Positive Luminal Breast Cancer |
|  | Proteins with Altered Expression in Breast Cancer |
|  | Breast Cancer |
|  | NOTCH1 Signaling in Breast Cancer |
|  | Endometrial Cancer |
|  | Proteins Involved in Endometrial Cancer |
|  | Proteins with Altered Expression in Endometrial Cancer |
|  | Local Estrogen Production in Endometriosis |
|  | Proteins Involved in Endometriosis |
|  | Angiogenesis in Endometriosis |
|  | Proteins with Altered Expression in Endometriosis |
|  | Coagulation Factors and PAR1/2 Receptors (F2R/F2RL1) in Endometriosis |
|  | Proteins Involved in Spontaneous Abortion |
|  | Proteins Involved in High-Grade Serous Ovarian Carcinoma |
|  | High-Grade Serous Ovarian Carcinoma |
|  | Ovarian Cancer |
|  | Proteins with Altered Expression in Ovarian Cancer |
|  | Proteins Involved in Cholesteatoma |
|  | IL6/JAK/STAT3 Signaling in Cholesteatoma |
|  | Cholesteatoma |
|  | HPV Infection and Cancer |
|  | HPV E6 and E7 Induced Disruption of TNFR/NF-kB Signaling in Keratinocytes |
|  | HPV E5 Regulates EDNRA and EGFR Signaling Pathway in Keratinocytes |
|  | Kruppel-like Factor 6 Signaling in Prostate Cancer |
|  | Androgen Receptor/Beta-2-Microglobulin Signaling in Prostate Cancer |
|  | Prostate Cancer |
|  | Proteins Involved in Prostate Cancer |
|  | Netrin-1 Signaling in Age-Related Macular Degeneration |
|  | Oxidative Stress, All-Trans-Retinal and Lipofuscin Toxicity in AMD |
|  | Proteins Involved in Age-Related Macular Degeneration |
|  | IL10/STAT3 Signaling in M2 Macrophage and Retinal Angiogenesis |
|  | ER Stress in Macular Degeneration |
|  | Genes with Mutations Associated with Age-Related Macular Degeneration |
|  | Proteins Involved in Cataract |
|  | HIF1A in Pathologic Ocular Neovascularization |
|  | VEGFA in Neovascular Glaucoma |
|  | Proteins with Altered Expression in Glaucoma |
|  | Proteins Involved in Noise-Induced Hearing Loss |
|  | Proteins with Altered Expression in Chronic Obstructive Pulmonary Disease |
|  | Proteins Involved in Chronic Obstructive Pulmonary Disease |
|  | VEGFA Low Expression Level in Chronic Bronchitis |
|  | VEGFA High Expression Level in Pulmonary Emphysema |
|  | Proteins Involved in Pulmonary Emphysema |
|  | Proteins Involved in Chronic Bronchitis |
|  | Proteins Involved in Erectile Dysfunction |
|  | Proteins Involved in Glomerulonephritis |
|  | Endothelial Cell Dysfunction in Glomerulonephritis |
|  | Podocyte Dysfunction in Glomerulonephritis |
|  | HMGB1 Expression Targets |
|  | S100B Expression Targets |
|  | Noradrenaline/Gs Expression Targets |
|  | Serotonin/Gs Expression Targets |
|  | Noreadrenaline/Gq Expression Targets |
|  | Acetylcholine Expression Targets |
|  | Serotonin/Gq Expression Targets |
|  | BMP6/ACVR2A Expression Targets |
|  | BMP2/BMPR2 Expression Targets |
|  | BMP7/ACVR2 Expression Targets |
|  | BMP4/BMPR2 Expression Targets |
|  | BMP7/BMPR2/ACVR2 Expression Targets |
|  | Elevated Receptors -> Expression Targets in Joint |
|  | Receptor Tyrosine Kinase Family -> Expression Targets in Bone |
|  | Elevated Receptors -> Expression Targets in Bone and Joint |
|  | Elevated Receptors -> Expression Targets in Bone |
|  | CD8 -> ATF/CREB/CREBBP Expression Targets |
|  | CTF1 Expression Targets |
|  | Elevated Receptors -> Expression Targets in Blood vessel |
|  | Elevated Receptors -> Expression Targets in Aorta |
|  | Elevated Receptors -> Expression Targets in Blood Vessel |
|  | CXCL1 Expression Targets |
|  | CXCL2 Expression Targets |
|  | CXCL12 Expression Targets |
|  | CCL3 Expression Targets |
|  | CCL3L1 Expression Target |
|  | CSF2 -> NF-kB Expression Targets |
|  | CSF2 -> STAT Expression Targets |
|  | LIF Expression Targets |
|  | EGF -> CTNN Expression Targets |
|  | EGF -> HIF1A Expression Targets |
|  | EGF -> AP-1/ATF Expression Targets |
|  | EGF -> CREB/CREBBP/ELK/SRF/MYC Expression Targets |
|  | EGF -> MEF/MYOD/NFATC Expression Targets |
|  | EGF -> STAT Expression Targets |
|  | Erythropoietin -> NF-kB Expression Targets |
|  | Erythropoietin -> AP-1/MYC/CREB Expression Targets |
|  | Erythropoietin -> STAT Expression Targets |
|  | Erythropoietin -> ELK/SRF Expression Targets |
|  | F2 -> AP-1/CREB/ELK/SRF/SP1 Expression Targets |
|  | F2 -> STAT1/NF-kB Expression Targets |
|  | PLG -> STAT1/NF-kB Expression Targets |
|  | FGF9 -> AP-1/CREB/MYC Expression Targets |
|  | FGF2 -> STAT Expression Targets |
|  | FGF1 -> AP-1/CREB/ELK/SRF/MYC Expression Targets |
|  | FGF7 -> RUNX Expression Targets |
|  | FGF1 -> STAT Expression Targets |
|  | FGF9 -> STAT Expression Targets |
|  | FGF9 -> RUNX Expression Targets |
|  | FGF7 -> AP-1/CREB/CREBBP/MYC Expression Targets |
|  | FGF2 -> AP-1/CREB/CREBBP/ELK/SRF/MYC Expression Targets |
|  | GCG Expression Targets |
|  | ADCYAP1 Expression Targets |
|  | FSHR Expression Targets |
|  | VIP Expression Targets |
|  | GH1/GHR -> NF-kB/ELK/SRF/MYC Expression Targets |
|  | GH1/GHR -> STAT Expression Targets |
|  | GH1/PRLR Expression Targets |
|  | HBEGF -> STAT Expression Targets |
|  | HBEGF -> AP-1/ATF Expression Targets |
|  | HBEGF -> EP300/ETS/ETV/SP1 Expression Targets |
|  | HBEGF -> HIF1A Expression Targets |
|  | HBEGF -> MEF/MYOD Expression Target |
|  | HBEGF -> CREB/MYC Expression Targets |
|  | HGF -> STAT Expression Targets |
|  | HGF -> AP-1/CREB/ELK/SRF/MYC Expression Targets |
|  | STAT3 and NFkB Activate Inflammation-Induced Tumorigenesis |
|  | IL23A and IL17A Provoke Cancer-Associated Inflammation |
|  | STAT3 Inhibits Maturation of Dendritic Cells and Facilitates Cancer Progression |
|  | Proteins with Altered Expression in Tumor-Promoting Inflammation |
|  | N2 Neutrophils in Tumor-Promoting Inflammation and Tumor Progression |
|  | Oxidized LDL/OLR1 Activates Cell Proliferation in Cancer |
|  | HIF1A in Vasculogenic Mimicry of Cancer |
|  | VEGFA Dependent Angiogenesis in Cancer |
|  | ANGPT/TEK Maintains Endothelial Cell Quiescence in Cancer |
|  | ANGPT/TEK Stimulates Endothelial Cell Migration in Cancer |
|  | Inhibition of THBS1 Activates Angiogenesis in Cancer |
|  | FBLN5 Potentiates Migration and Proliferation of Endothelial Cells in Cancer |
|  | HIF1A as Master Regulator of Angiogenesis in Cancer |
|  | Proteins with Altered Expression in Cancer Metastases |
|  | BSG (CD147) in Cancer Cells Motility, Invasion and Survival |
|  | Lactate as a Signaling Molecule in Cancer Cells |
|  | Proteins with Altered Expression in Cancer Metabolic Reprogramming |
|  | Tumor Infiltrating Macrophages in Cancer Progression and Immune Escape |
|  | IGF1 -> MEF/MYOD/MYOG Expression Targets |
|  | IGF1 -> CEBPA/FOXO1A Expression Targets |
|  | IGF2 -> STAT Expression Targets |
|  | IGF1 -> STAT Expression Targets |
|  | IGF2 -> CEBPA/FOXO1A Expression Targets |
|  | IGF2/MEF/MYOD Expression Targets |
|  | IGF2 -> HIF1A/MYC Expression Targets |
|  | IGF1 -> ELK/SRF/HIF1A/MYC/SREBF Expression Targets |
|  | INHBA/ACVR2/BMPR Expression Targets |
|  | INHBA/ACVR2/ACVR1 Expression Targets |
|  | Insulin -> STAT Expression Targets |
|  | Insulin -> CEBPA/CTNNB/FOXA/FOXO Expression Targets |
|  | Insulin -> MEF/MYOD Expression Targets |
|  | Insulin -> ELK/SRF/HIF1A/MYC/SREBF Expression Targets |
|  | CTGF -> AP-1/CREB/MYC Expression Targets |
|  | Fibrinogen Expression Targets |
|  | EGFR -> Expression Targets in Skin |
|  | Elevated Receptors -> Expression Targets in Skin |
|  | IFNA1/IFNR Expression Targets |
|  | IFNG/IFNR Expression Targets |
|  | IL13 Expression Targets |
|  | IL3 Expression Targets |
|  | IL6 Expression Targets |
|  | IL11 Expression Targets |
|  | Leptin -> STAT Expression Targets |
|  | Leptin -> ELK/SRF Expression Targets |
|  | Prostaglandin F Expression Targets |
|  | LPA Expression Targets |
|  | PAF/Gq -> AP-1/ATF1/CREB/ERK/SRF Expression Targets |
|  | Morphine Expression Targets |
|  | S1P Expression Targets |
|  | PGE1 Expression Targets |
|  | Elevated Receptors -> Expression Targets in Lymphoid System and Blood |
|  | GPCRs Family -> Expression Targets in Lymphoid System and Blood |
|  | Receptor Tyrosine Kinase Family -> Expression Targets in Lymphoid System and Blood |
|  | CD40 -> Expression Targets in Lymph Node |
|  | NRG1 -> MEF/MYOD Expression Targets |
|  | NRG1 -> EP300/ETS/ETV/SP1 Expression Targets |
|  | NRG1 -> CREB/CREBBP/ELK/SRF/MYC Expression Targets |
|  | NRG1/Catenin Expression Targets |
|  | NRG1 -> STAT Expression Targets |
|  | NRG1 -> AP-1/ATF Expression Targets |
|  | NRG1 -> HIF1A Expression Target |
|  | PTGER4 -> Expression Targets in Nerve Tissue |
|  | NTRK2 -> Expression Targets in Brain |
|  | NGF -> SMAD3/NF-kB Expression Targets |
|  | NGF -> AP-1/TP53/MYC Expression Targets |
|  | BDNF Expression Targets |
|  | ADAM17 Expression Targets |
|  | Adenosine Expression Targets |
|  | OSM/OSMR Expression Targets |
|  | PDGFB -> AP-1/CREB/MYC Expression Target |
|  | PDGFD -> AP-1 Expression Targets |
|  | PDGF -> AP-1/CREB/CREBBP/MYC Expression Targets |
|  | PDGF -> STAT Expression Targets |
|  | PDGFB -> STAT Expression Target |
|  | POMC Expression Targets |
|  | EDN1 Expression Targets |
|  | AGT -> CREB Expression Targets |
|  | AGT -> STAT Expression Targets |
|  | PRL/PRLR Expression Targets |
|  | PRL/GHR -> STAT Expression Targets |
|  | PRL/GHR -> NF/kB/ELK/SRF/MYC Expression Targets |
|  | CSF1 -> STAT Expression Targets |
|  | KITLG -> STAT Expression Targets |
|  | KITLG -> AP-1/CREB/CREBBP/MYC Expression Targets |
|  | EFNA1 -> STAT Expression Target |
|  | PAF Expression Targets |
|  | CSF1 -> AP-1/CREB/CREBBP/MYC Expression Targets |
|  | PGF -> AP-1/CREB/CREBBP/MYC Expression Targets |
|  | GDF5/BMPR2/ACVR2 Expression Targets |
|  | Elevated Receptors -> Expression Targets in Uterus |
|  | Elevated Receptors -> Expression Targets in Ear |
|  | HIF1 Signaling |
|  | Ras-GAP Regulation Signaling |
|  | VEGF Signaling |
|  | TGFA -> STAT Expression Targets |
|  | TGFA -> CTNNB/CTNND Expression Targets |
|  | TGFA -> HIF1A Expression Targets |
|  | TGFA -> MEF/MYOD/NFATC Expression Targets |
|  | TGFA/AP-1/ATF Expression Targets |
|  | TGFA -> CREB/CREBBP/ELK-SRF/MYC Expression Targets |
|  | TGFB2-TGFBR1 Expression Targets |
|  | TGFB3-TGFBR1 Expression Targets |
|  | TGFB1-TGFBR1 Expression Targets |
|  | TGFB3-TGFBR2 Expression Targets |
|  | TGFB2-TGFBR2 Expression Targets |
|  | TGFB1-TGFBR2 Expression Targets |
|  | TGFB1-TGFBR1/AP-1 Expression Targets |
|  | TGFB1-ACVRL1 Expression Targets |
|  | TNF -> NF-kB Expression Targets |
|  | TNF -> AP-1 Expression Targets |
|  | CD72 -> AP-1 Expression Targets |
|  | CD40LG -> STAT Expression Targets |
|  | CD40LG -> NF-kB/ELK/SRF -> CREB/NFATC Expression Targets |
|  | TNF -> STAT Expression Targets |
|  | Thrombopoietin -> AP-1/CREB/CREBBP/MYC Expression Targets |
|  | Thrombopoietin -> STAT Expression Targets |
|  | Thrombopoietin -> SP1 Expression Targets |
|  | TLR3 Expression Targets |
|  | TLR4 -> AP-1 Expression Targets |
|  | IL1B -> NO Expression Targets |
|  | IL1B -> PGE2 Expression Targets |
|  | TLR1 -> 2/6 Expression Targets |
|  | IL1B Expression Targets |
|  | TLR4 -> NF-kB/IRF Expression Targets |
|  | IL1A Expression Targets |
|  | ROS in Triggering Vascular Inflammation |
|  | VEGFA -> ATF/CREB/ELK-SRF Expression Targets |
|  | VEGFA -> NFATC Expression Targets |
|  | VEGFA -> STAT Expression Targets |
|  | VEGFA -> FOXO3A Expression Targets |
|  | VEGFA -> AP-1/CREBBP/MYC Expression Targets |
|  | VEGFA -> CTNNB/CTNND Expression Targets |
|  | VEGFA -> NCOR2 Expression Target |
|  | VEGFR -> CTNND Signaling |
|  | VEGFR -> CTNNB Signaling |
|  | VEGFR -> NFATC Signaling |
|  | VEGFR -> STAT Signaling |
|  | VEGFR -> AP-1/CREB/MYC Signaling |
|  | VEGFR -> FOXO3A Signaling |
|  | VEGFR -> ATF/CREB/ELK-SRF Signaling |
|  | WNT1 Expression Targets |
|  | WNT3A Expression Targets |
|  | WNT Canonical Signaling Expression Targets |
|  | pprarg neg, uknown targets, ps-positive |
|  | prarg neg,ukn expres targets, ps-positive |
|  | PPARG negative targets |
|  | pparg expr target\_regulators |
|  | Neighbors of disease exacerbation |
|  | Neighbors of tumor progression |
|  | Neighbors of cancer progression |
|  | Neighbors of inflammatory status |
|  | Neighbors of overall survival |
|  | Neighbors of disease severity |
|  | Neighbors of tumor size |
|  | Neighbors of lesion size |
|  | Neighbors of mortality |
|  | Neighbors of survival rate |
|  | Neighbors of infarct size |
|  | Neighbors of survival time |
|  | Neighbors of functional recovery |
|  | Neighbors of insulin sensitivity |
|  | Neighbors of bacterial load |
|  | Neighbors of cancer survival |
|  | Neighbors of graft survival |
|  | Neighbors of therapeutic efficacy |
|  | Neighbors of clinical stage |
|  | Neighbors of lethality |
|  | Neighbors of tumor regression |
|  | Neighbors of recurrence-free survival |
|  | Neighbors of disease-free survival |
|  | Neighbors of tumor response |
|  | Neighbors of macrophage count |
|  | Neighbors of long-term survival |
|  | Neighbors of treatment outcome |
|  | Neighbors of bone mass |
|  | Neighbors of body weight |
|  | Neighbors of body weight gain |
|  | Neighbors of cardiovascular risk |
|  | Neighbors of microvessel density |
|  | Neighbors of tumor recurrence |
|  | Neighbors of tumor promotion |
|  | Neighbors of glucose tolerance |
|  | Neighbors of morbidity |
|  | Neighbors of fibrosis progression |
|  | Neighbors of mortality rate |
|  | Neighbors of fat mass |
|  | Neighbors of exhaustion |
|  | Neighbors of bone mineral density |
|  | Neighbors of adverse outcome |
|  | Neighbors of radioresistance |
|  | Neighbors of cell density |
|  | Neighbors of cancer-specific survival |
|  | Neighbors of progression-free survival |
|  | Neighbors of apoptosis rate |
|  | Neighbors of early mortality |
|  | Neighbors of shear stress |
|  | Neighbors of blood pressure |
|  | Neighbors of leukocyte count |
|  | Neighbors of tumor grade |
|  | Neighbors of vascular reactivity |
|  | Neighbors of vascular leakage |
|  | Neighbors of body mass index |
|  | Neighbors of vascular density |
|  | Neighbors of muscle mass |
|  | Neighbors of cancer risk |
|  | Neighbors of cancer incidence |
|  | Neighbors of tumor aggressiveness |
|  | Neighbors of systolic blood pressure |
|  | Neighbors of tumor resistance |
|  | Neighbors of blood glucose |
|  | Neighbors of ejection fraction |
|  | Neighbors of median survival |
|  | Neighbors of renal hypertrophy |
|  | Neighbors of fasting plasma glucose |
|  | Neighbors of osteoclast number |
|  | Neighbors of cancer recurrence |
|  | Neighbors of T-cell count |
|  | Neighbors of antimetastatic activity |
|  | Neighbors of energy expenditure |
|  | Neighbors of glucose level |
|  | Neighbors of vascular tone |
|  | Neighbors of radiosensitivity |
|  | Neighbors of left ventricular ejection fraction |
|  | Neighbors of breast cancer risk |
|  | Neighbors of proliferation index |
|  | Neighbors of cell permeability |
|  | Neighbors of pregnancy outcome |
|  | Neighbors of neurological outcome |
|  | Neighbors of infarct area |
|  | Neighbors of fertility |
|  | Neighbors of recurrence rate |
|  | Neighbors of pathological response |
|  | Neighbors of skin thickness |
|  | Neighbors of insulin level |
|  | Neighbors of comorbidity |
|  | Neighbors of bone volume |
|  | Neighbors of fibroblast phenotype |
|  | Neighbors of fractional shortening |
|  | Neighbors of disease exacerbation |
|  | Neighbors of tumor progression |
|  | Neighbors of cancer progression |
|  | Neighbors of inflammatory status |
|  | Neighbors of overall survival |
|  | Neighbors of disease severity |
|  | Neighbors of tumor size |
|  | Neighbors of lesion size |
|  | Neighbors of mortality |
|  | Neighbors of survival rate |
|  | Neighbors of infarct size |
|  | Neighbors of survival time |
|  | Neighbors of functional recovery |
|  | Neighbors of insulin sensitivity |
|  | Neighbors of bacterial load |
|  | Neighbors of cancer survival |
|  | Neighbors of graft survival |
|  | Neighbors of therapeutic efficacy |
|  | Neighbors of clinical stage |
|  | Neighbors of lethality |
|  | Neighbors of tumor regression |
|  | Neighbors of recurrence-free survival |
|  | Neighbors of disease-free survival |
|  | Neighbors of tumor response |
|  | Neighbors of macrophage count |
|  | Neighbors of long-term survival |
|  | Neighbors of treatment outcome |
|  | Neighbors of bone mass |
|  | Neighbors of body weight |
|  | Neighbors of body weight gain |
|  | Neighbors of cardiovascular risk |
|  | Neighbors of microvessel density |
|  | Neighbors of tumor recurrence |
|  | Neighbors of tumor promotion |
|  | Neighbors of glucose tolerance |
|  | Neighbors of morbidity |
|  | Neighbors of fibrosis progression |
|  | Neighbors of mortality rate |
|  | Neighbors of fat mass |
|  | Neighbors of exhaustion |
|  | Neighbors of bone mineral density |
|  | Neighbors of adverse outcome |
|  | Neighbors of radioresistance |
|  | Neighbors of cell density |
|  | Neighbors of cancer-specific survival |
|  | Neighbors of progression-free survival |
|  | Neighbors of apoptosis rate |
|  | Neighbors of early mortality |
|  | Neighbors of shear stress |
|  | Neighbors of blood pressure |
|  | Neighbors of leukocyte count |
|  | Neighbors of tumor grade |
|  | Neighbors of vascular reactivity |
|  | Neighbors of vascular leakage |
|  | Neighbors of body mass index |
|  | Neighbors of vascular density |
|  | Neighbors of muscle mass |
|  | Neighbors of cancer risk |
|  | Neighbors of cancer incidence |
|  | Neighbors of tumor aggressiveness |
|  | Neighbors of systolic blood pressure |
|  | Neighbors of tumor resistance |
|  | Neighbors of blood glucose |
|  | Neighbors of ejection fraction |
|  | Neighbors of median survival |
|  | Neighbors of renal hypertrophy |
|  | Neighbors of fasting plasma glucose |
|  | Neighbors of osteoclast number |
|  | Neighbors of cancer recurrence |
|  | Neighbors of T-cell count |
|  | Neighbors of antimetastatic activity |
|  | Neighbors of energy expenditure |
|  | Neighbors of glucose level |
|  | Neighbors of vascular tone |
|  | Neighbors of radiosensitivity |
|  | Neighbors of left ventricular ejection fraction |
|  | Neighbors of breast cancer risk |
|  | Neighbors of proliferation index |
|  | Neighbors of cell permeability |
|  | Neighbors of pregnancy outcome |
|  | Neighbors of neurological outcome |
|  | Neighbors of infarct area |
|  | Neighbors of fertility |
|  | Neighbors of recurrence rate |
|  | Neighbors of pathological response |
|  | Neighbors of skin thickness |
|  | Neighbors of insulin level |
|  | Neighbors of comorbidity |
|  | Neighbors of bone volume |
|  | Neighbors of fibroblast phenotype |
|  | Neighbors of fractional shortening |
|  | Neighbors of cell development |
|  | Neighbors of immune response |
|  | Neighbors of cell population |
|  | Neighbors of T-cell development |
|  | Neighbors of inflammatory response |
|  | Neighbors of wound healing |
|  | Neighbors of angiogenesis |
|  | Neighbors of innate immune response |
|  | Neighbors of cell function |
|  | Neighbors of macrophage activation |
|  | Neighbors of cell formation |
|  | Neighbors of adaptive immune response |
|  | Neighbors of osteoclast development |
|  | Neighbors of epithelial to mesenchymal transition |
|  | Neighbors of immunity |
|  | Neighbors of cell proliferative response |
|  | Neighbors of chemotaxis |
|  | Neighbors of dendritic cell differentiation |
|  | Neighbors of cell survival |
|  | Neighbors of osteoclast differentiation |
|  | Neighbors of cell phenotype |
|  | Neighbors of T-cell proliferation |
|  | Neighbors of tissue remodeling |
|  | Neighbors of cell adhesion |
|  | Neighbors of T-cell activation |
|  | Neighbors of endothelial cell proliferation |
|  | Neighbors of fibrogenesis |
|  | Neighbors of tumor growth |
|  | Neighbors of dendritic cell development |
|  | Neighbors of tissue repair |
|  | Neighbors of cell count |
|  | Neighbors of cell infiltration |
|  | Neighbors of cell interaction |
|  | Neighbors of SMC proliferation |
|  | Neighbors of lumen formation |
|  | Neighbors of endothelial cell migration |
|  | Neighbors of adipogenesis |
|  | Neighbors of lipid storage |
|  | Neighbors of cell damage |
|  | Neighbors of pregnancy |
|  | Neighbors of monocyte differentiation |
|  | Neighbors of aging |
|  | Neighbors of cell motility |
|  | Neighbors of bone resorption |
|  | Neighbors of monocyte migration |
|  | Neighbors of fibroblast proliferation |
|  | Neighbors of adipocyte differentiation |
|  | Neighbors of macrophage migration |
|  | Neighbors of microglial activation |
|  | Neighbors of neutrophil recruitment |
|  | Neighbors of regeneration |
|  | Neighbors of cell migration |
|  | Neighbors of cancer growth |
|  | Neighbors of stem cell proliferation |
|  | Neighbors of smooth muscle cell migration |
|  | Neighbors of hepatic regeneration |
|  | Neighbors of transendothelial migration |
|  | Neighbors of phagocytosis |
|  | Neighbors of immune system activation |
|  | Neighbors of leukocyte migration |
|  | Neighbors of leukocyte recruitment |
|  | Neighbors of osteoclast formation |
|  | Neighbors of monocyte recruitment |
|  | Neighbors of cell invasion |
|  | Neighbors of heart function |
|  | Neighbors of hemopoiesis |
|  | Neighbors of bone remodeling |
|  | Neighbors of ossification |
|  | Neighbors of T-cell function |
|  | Neighbors of immune system function |
|  | Neighbors of lung development |
|  | Neighbors of autophagy |
|  | Neighbors of monocyte adhesion |
|  | Neighbors of transcription activation |
|  | Neighbors of senescence |
|  | Neighbors of T-cell response |
|  | Neighbors of tumor immunity |
|  | Neighbors of hepatocyte proliferation |
|  | Neighbors of cell transdifferentiation |
|  | Neighbors of neuroprotection |
|  | Neighbors of stem cell differentiation |
|  | Neighbors of endothelial cell function |
|  | Neighbors of neuronal death |
|  | Neighbors of cell growth |
|  | Neighbors of ROS generation |
|  | Neighbors of epithelial cell proliferation |
|  | Neighbors of first trimester pregnancy |
|  | Neighbors of cancer cell growth |
|  | Neighbors of sensitization |
|  | Neighbors of hemato-encephalic barrier |
|  | Neighbors of macrophage function |
|  | Neighbors of cellular immune response |
|  | Neighbors of cellular senescence |
|  | Neighbors of colony formation |
|  | Neighbors of macrophage differentiation |
|  | Neighbors of cell differentiation |
|  | Neighbors of keratinocyte proliferation |
|  | Neighbors of stem cell migration |
|  | Neighbors of life span |
|  | Neighbors of cell homeostasis |
|  | Neighbors of psoriasis |
|  | Neighbors of inflammatory disease |
|  | Neighbors of rheumatoid arthritis |
|  | Neighbors of atherosclerosis |
|  | Neighbors of fibrosis |
|  | Neighbors of colitis |
|  | Neighbors of arthritis |
|  | Neighbors of inflammatory bowel disease |
|  | Neighbors of injury |
|  | Neighbors of atherogenesis |
|  | Neighbors of vascular remodeling |
|  | Neighbors of metastasis |
|  | Neighbors of carcinogenesis |
|  | Neighbors of multiple sclerosis |
|  | Neighbors of chronic inflammation |
|  | Neighbors of inflammation |
|  | Neighbors of tumor microenvironment |
|  | Neighbors of pathological angiogenesis |
|  | Neighbors of obesity |
|  | Neighbors of vascular disease |
|  | Neighbors of diabetes mellitus |
|  | Neighbors of asthma |
|  | Neighbors of pneumonia |
|  | Neighbors of hyperplasia |
|  | Neighbors of osteoarthritis |
|  | Neighbors of liver fibrosis |
|  | Neighbors of chronic obstructive pulmonary disease |
|  | Neighbors of myocardial infarction |
|  | Neighbors of leukocyte infiltration |
|  | Neighbors of reperfusion injury |
|  | Neighbors of airway inflammation |
|  | Neighbors of hepatitis |
|  | Neighbors of insulin resistance |
|  | Neighbors of infection |
|  | Neighbors of vascular injury |
|  | Neighbors of renal injury |
|  | Neighbors of preeclampsia |
|  | Neighbors of macrophage infiltration |
|  | Neighbors of liver injury |
|  | Neighbors of neuroinflammation |
|  | Neighbors of sepsis |
|  | Neighbors of death |
|  | Neighbors of type 1 diabetes |
|  | Neighbors of encephalomyelitis |
|  | Neighbors of autoimmune disease |
|  | Neighbors of ischemic stroke |
|  | Neighbors of dermatitis |
|  | Neighbors of neutrophil infiltration |
|  | Neighbors of heart disease |
|  | Neighbors of diabetic nephropathy |
|  | Neighbors of lung injury |
|  | Neighbors of immunopathology |
|  | Neighbors of systemic sclerosis |
|  | Neighbors of hepatocellular carcinoma |
|  | Neighbors of acute lung injury |
|  | Neighbors of gastric cancer |
|  | Neighbors of atherosclerotic plaque |
|  | Neighbors of experimental autoimmune encephalomyelitis |
|  | Neighbors of colorectal cancer |
|  | Neighbors of cardiac remodeling |
|  | Neighbors of breast cancer |
|  | Neighbors of ischemia |
|  | Neighbors of systemic lupus erythematosus |
|  | Neighbors of melanoma |
|  | Neighbors of collagen-induced arthritis |
|  | Neighbors of cartilage degeneration |
|  | Neighbors of interstitial fibrosis |
|  | Neighbors of neoplasm invasion |
|  | Neighbors of kidney disease |
|  | Neighbors of heart failure |
|  | Neighbors of lung disease |
|  | Neighbors of pulmonary fibrosis |
|  | Neighbors of cardiovascular disease |
|  | Neighbors of stroke |
|  | Neighbors of cancer |
|  | Neighbors of cerebral ischemia |
|  | Neighbors of type 2 diabetes |
|  | Neighbors of neoplasm |
|  | Neighbors of endometriosis |
|  | Neighbors of neointima |
|  | Neighbors of vasculitis |
|  | Neighbors of atrophy |
|  | Neighbors of diet-induced obesity |
|  | Neighbors of respiratory hypersensitivity |
|  | Neighbors of myocardial fibrosis |
|  | Neighbors of synovitis |
|  | Neighbors of coronary artery disease |
|  | Neighbors of rupture |
|  | Neighbors of endothelial cell dysfunction |
|  | Neighbors of liver disease |
|  | Neighbors of prostate cancer |
|  | Neighbors of hypertrophy |
|  | Neighbors of osteolysis |
|  | Neighbors of ovarian cancer |
|  | Neighbors of pancreatic cancer |
|  | Neighbors of hypertension |
|  | Neighbors of intimal hyperplasia |
|  | Neighbors of allograft rejection |
|  | Neighbors of proteinuria |
|  | Neighbors of epithelial cell |
|  | Neighbors of keratinocyte |
|  | Neighbors of vascular smooth muscle cell |
|  | Neighbors of umbilical vein endothelial cell |
|  | Neighbors of monocyte |
|  | Neighbors of endometrial stromal cell |
|  | Neighbors of alveolar macrophage |
|  | Neighbors of synoviocyte |
|  | Neighbors of macrophage |
|  | Neighbors of adipocyte |
|  | Neighbors of activated monocyte |
|  | Neighbors of fibroblast |
|  | Neighbors of airway epithelial cell |
|  | Neighbors of PBMC |
|  | Neighbors of stromal cell |
|  | Neighbors of airway smooth muscle cell |
|  | Neighbors of osteoblast |
|  | Neighbors of pneumocyte |
|  | Neighbors of endometrium cell |
|  | Neighbors of microglia |
|  | Neighbors of trophoblast |
|  | Neighbors of intestine epithelium cell |
|  | Neighbors of mononuclear cell |
|  | Neighbors of hepatic stellate cell |
|  | Neighbors of hepatocyte |
|  | Neighbors of decidual cell |
|  | Neighbors of kidney mesangial cell |
|  | Neighbors of endothelial cell |
|  | Neighbors of dendritic cell |
|  | Neighbors of Kuppfer cell |
|  | Neighbors of proadipocyte |
|  | Neighbors of kidney tubule cell |
|  | Neighbors of inflammatory cell |
|  | Neighbors of bone marrow macrophage |
|  | Neighbors of peritoneal macrophage |
|  | Neighbors of osteoclast |
|  | Neighbors of neutrophil |
|  | Neighbors of smooth muscle myocyte |
|  | Neighbors of astrocyte |
|  | Neighbors of bone marrow derived dendritic cell |
|  | Neighbors of lung fibroblast |
|  | Neighbors of activated T-cell |
|  | Neighbors of mesothelial cell |
|  | Neighbors of mononuclear phagocyte |
|  | Neighbors of capillary endothelial cell |
|  | Neighbors of immunocompetent cell |
|  | Neighbors of granulocyte |
|  | Neighbors of cardiac myocyte |
|  | Neighbors of cancer-associated fibroblast |
|  | Neighbors of myeloid cell |
|  | Neighbors of mesenchymal stroma cell |
|  | Neighbors of corneal epithelial cell |
|  | Neighbors of cytotrophoblast |
|  | Neighbors of stem cell |
|  | Neighbors of tumor infiltrating macrophage |
|  | Neighbors of sinusoidal cell |
|  | Neighbors of B-cell |
|  | Neighbors of granulosa cell |
|  | Neighbors of leukocyte |
|  | Neighbors of chondrocyte |
|  | Neighbors of insulin-secreting cell |
|  | Neighbors of mesenchymal cell |
|  | Neighbors of T-cell |
|  | Neighbors of M1 macrophage |
|  | Neighbors of myofibroblast |
|  | Neighbors of pancreatic stellate cell |
|  | Neighbors of mast cell |
|  | Neighbors of bone marrow stromal cell |
|  | Neighbors of spleen cell |
|  | Neighbors of brain capillary endothelial cell |
|  | Neighbors of neuroglia |
|  | Neighbors of thyroid epithelial cell |
|  | Neighbors of aortic smooth muscle cell |
|  | Neighbors of hematopoietic cell |
|  | Neighbors of podocyte |
|  | Neighbors of mammary epithelial cell |
|  | Neighbors of pericyte |
|  | Neighbors of epidermis cell |
|  | Neighbors of bone marrow derived mast cell |
|  | Neighbors of phagocyte |
|  | Neighbors of kidney cell |
|  | Neighbors of syncytiotrophoblast |
|  | Neighbors of lymphocyte |
|  | Neighbors of decidual natural killer cell |
|  | Neighbors of thymic epithelial cell |
|  | Neighbors of brain cell |
|  | Neighbors of M2 macrophage |
|  | Neighbors of peritoneum cell |
|  | Neighbors of lymphoid cell |
|  | Neighbors of retina cell |
|  | Neighbors of blood cell |
|  | Neighbors of skin cell |
|  | Neighbors of luteal cell |
|  | Neighbors of peripheral lymphocyte |
|  | Neighbors of activated B-cell |
|  | Neighbors of lung |
|  | Neighbors of liver |
|  | Neighbors of kidney |
|  | Neighbors of skin |
|  | Neighbors of intestine |
|  | Neighbors of lymph node |
|  | Neighbors of colon |
|  | Neighbors of airway |
|  | Neighbors of blood vessel |
|  | Neighbors of heart |
|  | Neighbors of aorta |
|  | Neighbors of gastrointestinal tract |
|  | Neighbors of microvessel |
|  | Neighbors of bone |
|  | Neighbors of placenta |
|  | Neighbors of carotid artery |
|  | Neighbors of synovial membrane |
|  | Neighbors of uterus |
|  | Neighbors of esophagus |
|  | Neighbors of periodontium |
|  | Neighbors of retina |
|  | Neighbors of brain |
|  | Neighbors of ovary |
|  | Neighbors of breast |
|  | Neighbors of blood vessel wall |
|  | Neighbors of stomach |
|  | Neighbors of islets of Langerhans |
|  | Neighbors of artery |
|  | Neighbors of cardiovascular system |
|  | Neighbors of pancreas |
|  | Neighbors of prostate |
|  | Neighbors of central nervous system |
|  | Neighbors of kidney glomerulus |
|  | Neighbors of lymphatic system |
|  | Neighbors of colorectal region |
|  | Neighbors of decidua |
|  | Neighbors of amnion |
|  | Neighbors of immune system |
|  | Neighbors of peritoneum |
|  | Neighbors of alveolus |
|  | Neighbors of coronary artery |
|  | Neighbors of left ventricle |
|  | Neighbors of uterine cervix |
|  | Neighbors of omentum |
|  | Neighbors of vein |
|  | Neighbors of arteriole |
|  | Neighbors of villus |
|  | Neighbors of bronchus |
|  | Neighbors of glands |
|  | Neighbors of nose |
|  | Neighbors of eye |
|  | Neighbors of cornea |
|  | Neighbors of hippocampus |
|  | Neighbors of intervertebral disc |
|  | Neighbors of mammary gland |
|  | Neighbors of vitreous body |
|  | Neighbors of cerebral cortex |
|  | Neighbors of brain blood vessel |
|  | Neighbors of bladder |
|  | Neighbors of nervous system |
|  | Neighbors of retina blood vessel |
|  | Neighbors of mesentery |
|  | Neighbors of nasopharynx |
|  | Neighbors of cervical lymph node group |
|  | Neighbors of periodontal ligament |
|  | Neighbors of trachea |
|  | Neighbors of corpus luteum of ovary |
|  | Neighbors of nerve |
|  | Neighbors of endocrine system |
|  | Neighbors of lower extremity |
|  | Neighbors of small intestine |
|  | Neighbors of aggregated lymphoid follicle of small intestine |
|  | Neighbors of knee joint |
|  | Neighbors of lymphatic vessels |
|  | Neighbors of pleura |
|  | Neighbors of Fallopian tube |
|  | Neighbors of hair follicle |
|  | Neighbors of NF-kB family |
|  | Neighbors of oxidized LDL |
|  | Neighbors of JNK |
|  | Neighbors of IL1 family |
|  | Neighbors of PPAR |
|  | Neighbors of Jun/Fos |
|  | Neighbors of NFKBI |
|  | Neighbors of PKC |
|  | Neighbors of PI3K |
|  | Neighbors of STAT family |
|  | Neighbors of mitogen-activated protein kinase |
|  | Neighbors of IFNAR ligand |
|  | Neighbors of transforming growth factor |
|  | Neighbors of TLR |
|  | Neighbors of cytokine |
|  | Neighbors of interferon |
|  | Neighbors of interleukin |
|  | Neighbors of JAK |
|  | Neighbors of MEK1/2 |
|  | Neighbors of PDGF |
|  | Neighbors of Ras GTPase |
|  | Neighbors of ERK1/2 |
|  | Neighbors of growth factor |
|  | Neighbors of PKA |
|  | Neighbors of ovalbumin |
|  | Neighbors of LDL |
|  | Neighbors of NF-AT family |
|  | Neighbors of beta adrenoceptor |
|  | Neighbors of chorionic gonadotropin |
|  | Neighbors of IKBK |
|  | Neighbors of adipokine |
|  | Neighbors of protein tyrosine kinase |
|  | Neighbors of NAD(P)H oxidase |
|  | Neighbors of IgG |
|  | Neighbors of SMAD subfamily |
|  | Neighbors of AMPK |
|  | Neighbors of LXR |
|  | Neighbors of HDL |
|  | Neighbors of p38 |
|  | Neighbors of inflammatory cytokine |
|  | Neighbors of estrogen receptor |
|  | Neighbors of T-cell receptor |
|  | Neighbors of histone deacetylase |
|  | Neighbors of PDGF-BB |
|  | Neighbors of IL1R |
|  | Neighbors of HIF-1 |
|  | Neighbors of GPCR |
|  | Neighbors of IL12 |
|  | Neighbors of Notch |
|  | Neighbors of prostaglandin-endoperoxide synthase |
|  | Neighbors of heat shock protein 90 |
|  | Neighbors of fibrinogen |
|  | Neighbors of aldehyde reductase |
|  | Neighbors of mitogen-activated protein kinase kinase |
|  | Neighbors of interleukin-35 |
|  | Neighbors of TSH |
|  | Neighbors of Ang II receptor |
|  | Neighbors of endothelin |
|  | Neighbors of NOS |
|  | Neighbors of superoxide dismutase |
|  | Neighbors of proteasome endopeptidase complex |
|  | Neighbors of class nuclear receptor with C4 zinc fingers |
|  | Neighbors of non-coding RNA |
|  | Neighbors of histone H3 |
|  | Neighbors of STAT5 |
|  | Neighbors of alpha2 adrenoceptor |
|  | Neighbors of vitronectin receptor (integrin) |
|  | Neighbors of retinoid-X receptor subfamily |
|  | Neighbors of SAPK |
|  | Neighbors of WNT |
|  | Neighbors of gonadotropins |
|  | Neighbors of immunoglobulin |
|  | Neighbors of PP2B |
|  | Neighbors of phospholipase |
|  | Neighbors of AHR subfamily |
|  | Neighbors of ITG |
|  | Neighbors of endostatins |
|  | Neighbors of relaxin |
|  | Neighbors of TLR1/2 heterodimer |
|  | Neighbors of FSH |
|  | Neighbors of TORC1 |
|  | Neighbors of angiotensin receptor |
|  | Neighbors of RA receptor |
|  | Neighbors of histone |
|  | Neighbors of Rho kinase |
|  | Neighbors of PDGF receptor |
|  | Neighbors of neuropeptides |
|  | Neighbors of pancreatic elastase |
|  | Neighbors of histone acetyltransferase |
|  | Neighbors of adrenoceptor |
|  | Neighbors of phosphodiesterase |
|  | Neighbors of caspase |
|  | Neighbors of deacetylase |
|  | Neighbors of TLR4/Myd88 |
|  | Neighbors of TNF-alpha receptor |
|  | Model of PPARG signaling in psoriasis |
|  | PPARs and anxiety common targets |
|  | Amygdaloid expressed genes |
|  | IFN signaling in alveolar cells |
|  | Overview. Diseases of circulatory system (chapter 8) |
|  | Overview. Diseases of the skin (chapter 11) |
|  | Overview. Diseases of the eye (chapter 6) |
|  | Overview. Diseases of the respiratory (chapter 9) |
|  | Overview. Diseases of the genitourinary system (chapter 13) |
|  | 2\_Interleukin-17 and interleukin-22 signaling in psoriasis |
|  | 1\_TNF and IL1B provoke ECM degradation in osteoarthritis |
|  | 1\_Local estrogen production stimulates ectopic endometrium proliferation |
|  | 3\_Enhanced angiogenesis in endometriosis |
|  | 2\_Oxidative stress, all-trans-retinal, and lipofuscin toxicity in agerelated macular degeneration |
|  | 3\_Endoplasmic reticulum stress in age-related macular degeneration |
|  | 1\_Endothelial cells stimulate vasoconstriction and thrombosis in pulmonary hypertension |
|  | 2\_3\_Eosinophilia and neutrophilia in asthma: Neutrophil chemotaxis and activation |
|  | 2\_2\_Eosinophilia and neutrophilia in asthma: eosinophil apoptosis |
|  | 4\_1\_Polymorphisms associated with inflammatory bowel diseases |
|  | 3\_Human papilloma virus E4 and E5 proteins in viral reproduction |
|  | 5\_Human papilloma virus E6 and E7 proteins promote epithelial cell proliferation |
|  | 1\_Microangiopathy in diabetic neuropathy |
|  | 2\_Mutations in SOD1 and associated proteins cause motor neuron death in familial ALS |
|  | drug-target pairs direct regulation |
|  | drug-target moltransport |
|  | New Combined Pathway |
|  | Interleukin-17 and interleukin-22 signaling in psoriasis |

---

|  |  |
| --- | --- |
| Group | Genes with Mutations Associated with Psoriasis |
|  | Genes with Polymorphisms Associated with Migraine |
|  | Genes with Mutations Associated with Endometriosis |
|  | Genes with Susceptibility Loci Associated with Age-Related Macular Degeneration |
|  | Chemokines, Growth Factors and Receptors Involved in Cancer Cells Chemotaxis |

---

|  |  |
| --- | --- |
| Source | GO component |
|  | curated |

---

|  |  |
| --- | --- |
| MedScan ID | 7422 |

---

|  |  |
| --- | --- |
| LocusLink ID | 7422 |
|  | 22339 |
|  | 83785 |
|  | 558154 |
|  | 281572 |
|  | 797569 |
|  | 114601 |

---

|  |  |
| --- | --- |
| Alias | Vascular endothelial growth factor 121 |
|  | VEGF120 |
|  | VEGF121 |
|  | VEGF164 |
|  | vascular endothelial growth factor variant |
|  | vascular endothelial growth factor |
|  | RP1-261G23.1 |
|  | MGC70609 |
|  | OTTMUSP00000017463 |
|  | OTTHUMP00000165987 |
|  | vascular endothelial cell growth factor |
|  | vascular endothelial growth factors |
|  | OTTMUSP00000022244 |
|  | vascular permeability factor |
|  | OTTMUSP00000022243 |
|  | VEGF-A |
|  | vascular endothelial cells growth factor |
|  | VPF |
|  | vasculotropin |
|  | vascular endothelial growth factor isoform VEGF165 |
|  | VEGF188 |
|  | vascular endothelial growth factor A |
|  | OTTHUMP00000224107 |
|  | VEGFA |
|  | OTTMUSP00000017464 |
|  | vascular endothelial growth factor A121 |
|  | GNB:37659 |
|  | OTTMUSP00000022245 |
|  | VEGF |
|  | VEGF165 |
|  | VEGF111 |
|  | vascular endothelial growth factor A165 |
|  | MVCD1 |
|  | Vascular endothelial growth factor precursor |
|  | Vascular permeability factor |
|  | VEGF164 protein |
|  | riken cdna g430002g23 gene |
|  | g430002g23rik |
|  | LOC558154 |
|  | DKEY-14D8.3 |
|  | chromosome |
|  | scaffold |
|  | si:dkey-14d8.3 |
|  | LOC797569 |
|  | similar to vascular endothelial growth factor |

---

|  |  |
| --- | --- |
| GO ID | 0042056 |
|  | 0005125 |
|  | 0050840 |
|  | 0001968 |
|  | 0008083 |
|  | 0008201 |
|  | 0005161 |
|  | 0046982 |
|  | 0042803 |
|  | 0048018 |
|  | 0043183 |
|  | 0043184 |
|  | 0005172 |
|  | 0038190 |
|  | 0032147 |
|  | 0001525 |
|  | 0048844 |
|  | 0001569 |
|  | 0048754 |
|  | 0048593 |
|  | 0048739 |
|  | 0060948 |
|  | 0048469 |
|  | 0002042 |
|  | 0071456 |
|  | 0035924 |
|  | 0097533 |
|  | 0071679 |
|  | 0060982 |
|  | 0003169 |
|  | 0019221 |
|  | 0071542 |
|  | 0035767 |
|  | 0030855 |
|  | 0042462 |
|  | 0003007 |
|  | 0001701 |
|  | 0050930 |
|  | 0001822 |
|  | 0007595 |
|  | 0030324 |
|  | 0036303 |
|  | 0030225 |
|  | 0060749 |
|  | 0007498 |
|  | 0030224 |
|  | 0097475 |
|  | 1903392 |
|  | 0043066 |
|  | 2000048 |
|  | 0043154 |
|  | 1903141 |
|  | 0010629 |
|  | 0000122 |
|  | 0007399 |
|  | 0003151 |
|  | 0001541 |
|  | 0002576 |
|  | 0050918 |
|  | 0032793 |
|  | 0043406 |
|  | 0045766 |
|  | 0048842 |
|  | 0043536 |
|  | 1903589 |
|  | 0090190 |
|  | 0045785 |
|  | 0051781 |
|  | 0030335 |
|  | 0090050 |
|  | 0008284 |
|  | 0038091 |
|  | 0051272 |
|  | 0120162 |
|  | 0038033 |
|  | 0010595 |
|  | 0001938 |
|  | 0050679 |
|  | 0051894 |
|  | 0010628 |
|  | 1901727 |
|  | 0002687 |
|  | 0060754 |
|  | 0002053 |
|  | 0002052 |
|  | 1900745 |
|  | 0033138 |
|  | 1900086 |
|  | 0050731 |
|  | 0050927 |
|  | 0031954 |
|  | 0031334 |
|  | 0090037 |
|  | 1903572 |
|  | 0001934 |
|  | 0002092 |
|  | 1902336 |
|  | 1903672 |
|  | 0045944 |
|  | 0061419 |
|  | 0042531 |
|  | 0030949 |
|  | 0043117 |
|  | 0031077 |
|  | 0060319 |
|  | 0008360 |
|  | 0010749 |
|  | 0090259 |
|  | 0006357 |
|  | 0061418 |
|  | 0001666 |
|  | 0002040 |
|  | 0043129 |
|  | 0035148 |
|  | 0048010 |
|  | 0038084 |
|  | 0001570 |
|  | 0009986 |
|  | 0005913 |
|  | 0005737 |
|  | 0031012 |
|  | 0005576 |
|  | 0005615 |
|  | 0016020 |
|  | 0031093 |
|  | 0030141 |
|  | 0019838 |
|  | 0038191 |
|  | 0005102 |
|  | 0042088 |
|  | 0060978 |
|  | 0002575 |
|  | 0001568 |
|  | 0002043 |
|  | 0048514 |
|  | 0001974 |
|  | 0060346 |
|  | 0061430 |
|  | 0060326 |
|  | 0030154 |
|  | 0016477 |
|  | 0008283 |
|  | 0043542 |
|  | 0048873 |
|  | 0048286 |
|  | 0060426 |
|  | 0001946 |
|  | 0048255 |
|  | 0007275 |
|  | 0045779 |
|  | 0022408 |
|  | 0010719 |
|  | 2001237 |
|  | 0045599 |
|  | 0043524 |
|  | 1901215 |
|  | 0043069 |
|  | 0048666 |
|  | 2000573 |
|  | 0070374 |
|  | 1901492 |
|  | 0045669 |
|  | 0042327 |
|  | 0051897 |
|  | 1902966 |
|  | 0009967 |
|  | 0048661 |
|  | 0045601 |
|  | 0040008 |
|  | 1901532 |
|  | 0097601 |
|  | 0001944 |
|  | 0005604 |
|  | 0005634 |
|  | 0005886 |
|  | 0031982 |
|  | 0007568 |
|  | 0071549 |
|  | 0035690 |
|  | 0044344 |
|  | 0007565 |
|  | 0030212 |
|  | 0010469 |
|  | 0009409 |
|  | 0071548 |
|  | 0032355 |
|  | 0051593 |
|  | 0070482 |
|  | 0032570 |
|  | 0033189 |
|  | 0061304 |
|  | 0042060 |
|  | 0043185 |
|  | 0040007 |
|  | 0042517 |
|  | 0030823 |
|  | 0005578 |
|  | 0031988 |
|  | 0007596 |
|  | 2000273 |
|  | 0030168 |
|  | 1902533 |
|  | 0007610 |
|  | 2001028 |
|  | 0033554 |
|  | 0005515 |
|  | 0043498 |
|  | 0006916 |
|  | 0010740 |
|  | 0048008 |
|  | 0071445 |
|  | 0009968 |
|  | 0006950 |
|  | 0000074 |
|  | 0005786 |
|  | 0048595 |
|  | 0007165 |

---

|  |  |
| --- | --- |
| KEGG ID | hsa:7422 |
|  | mmu:22339 |
|  | rno:83785 |

---

|  |  |
| --- | --- |
| MedLine Reference | 17255303 |
|  | 12660426 |
|  | 17178724 |
|  | 16005848 |
|  | 15047611 |
|  | 11986311 |
|  | 14998923 |
|  | 16906455 |
|  | 12591731 |
|  | 10364225 |
|  | 15173661 |
|  | 11847008 |
|  | 9665459 |
|  | 9846489 |
|  | 14691147 |
|  | 11751905 |
|  | 15705790 |
|  | 15568021 |
|  | 16166239 |
|  | 17148604 |
|  | 16516857 |
|  | 15745839 |
|  | 17638880 |
|  | 16271941 |
|  | 17442277 |
|  | 17319747 |
|  | 10409677 |
|  | 17008323 |
|  | 16116481 |
|  | 15509779 |
|  | 12466851 |
|  | 10433823 |
|  | 12545153 |
|  | 12917333 |
|  | 8632007 |
|  | 12666706 |
|  | 15515055 |
|  | 15970595 |
|  | 15094037 |
|  | 17003502 |
|  | 9792673 |
|  | 15338501 |
|  | 15705785 |
|  | 15249212 |
|  | 16291795 |
|  | 14570917 |
|  | 15640157 |
|  | 11921018 |
|  | 11897712 |
|  | 16179809 |
|  | 1791831 |
|  | 11897714 |
|  | 12087404 |
|  | 15188178 |
|  | 16328781 |
|  | 12127077 |
|  | 12370313 |
|  | 14722611 |
|  | 16999951 |
|  | 11084652 |
|  | 12890905 |
|  | 16914267 |
|  | 15459110 |
|  | 16513643 |
|  | 16152587 |
|  | 12517803 |
|  | 16224065 |
|  | 12428103 |
|  | 12239588 |
|  | 12213878 |
|  | 15458509 |
|  | 16636650 |
|  | 11777931 |
|  | 12213874 |
|  | 14555767 |
|  | 11796824 |
|  | 16484238 |
|  | 16002046 |
|  | 12505748 |
|  | 15714497 |
|  | 10229225 |
|  | 15262905 |
|  | 12684689 |
|  | 15883642 |
|  | 16024519 |
|  | 12087467 |
|  | 16891801 |
|  | 14644771 |
|  | 17428830 |
|  | 16440308 |
|  | 16543245 |
|  | 15003169 |
|  | 16543242 |
|  | 15453096 |
|  | 9212753 |
|  | 16702604 |
|  | 15178644 |
|  | 14714296 |
|  | 11728162 |
|  | 12085210 |
|  | 15979997 |
|  | 12444056 |
|  | 12546587 |
|  | 9689915 |
|  | 14644752 |
|  | 12800090 |
|  | 10704390 |
|  | 15312247 |
|  | 16678147 |
|  | 10952892 |
|  | 17296185 |
|  | 9716565 |
|  | 17084400 |
|  | 17293480 |
|  | 15716406 |
|  | 17498737 |
|  | 15754021 |
|  | 16775174 |
|  | 11961297 |
|  | 15131073 |
|  | 12637571 |
|  | 15084712 |
|  | 17259494 |
|  | 12631117 |
|  | 11042159 |
|  | 11290536 |
|  | 12452000 |
|  | 16508738 |
|  | 14759416 |
|  | 14654077 |
|  | 16932349 |
|  | 17417650 |
|  | 9778514 |
|  | 12208766 |
|  | 11437453 |
|  | 17082651 |
|  | 12391545 |
|  | 9446618 |
|  | 15126581 |
|  | 11877669 |
|  | 10328919 |
|  | 10585578 |
|  | 16880268 |
|  | 15669079 |
|  | 16924371 |
|  | 12574959 |
|  | 16778084 |
|  | 17332426 |
|  | 14977835 |
|  | 11352659 |
|  | 12039075 |
|  | 12590138 |
|  | 15774498 |
|  | 16585124 |
|  | 15638384 |
|  | 16778080 |
|  | 17289883 |
|  | 15474081 |
|  | 12124351 |
|  | 14702039 |
|  | 16488441 |
|  | 16799060 |
|  | 15734877 |
|  | 12716475 |
|  | 16375885 |
|  | 12215337 |
|  | 11839635 |
|  | 15809353 |
|  | 16410078 |
|  | 17360578 |
|  | 16441660 |
|  | 15050452 |
|  | 15521009 |
|  | 15247002 |
|  | 16973934 |
|  | 17374440 |
|  | 14728692 |
|  | 16421229 |
|  | 15470196 |
|  | 11784713 |
|  | 10021335 |
|  | 16887829 |
|  | 14699103 |
|  | 15063765 |
|  | 14604996 |
|  | 12058259 |
|  | 9401819 |
|  | 7525053 |
|  | 17537803 |
|  | 17334372 |
|  | 15742396 |
|  | 15947259 |
|  | 12631341 |
|  | 10067980 |
|  | 7681362 |
|  | 17249570 |
|  | 14634801 |
|  | 10748121 |
|  | 15326102 |
|  | 14712484 |
|  | 17001321 |
|  | 15342781 |
|  | 16570043 |
|  | 15254682 |
|  | 16263579 |
|  | 17237399 |
|  | 16887817 |
|  | 11267659 |
|  | 16467091 |
|  | 17203176 |
|  | 16596190 |
|  | 16769575 |
|  | 8090202 |
|  | 16000382 |
|  | 17013089 |
|  | 16105884 |
|  | 14512169 |
|  | 17464208 |
|  | 16398404 |
|  | 12800229 |
|  | 17289872 |
|  | 16239224 |
|  | 17543159 |
|  | 15556999 |
|  | 16132039 |
|  | 16521228 |
|  | 12393542 |
|  | 10495271 |
|  | 2479987 |
|  | 10066377 |
|  | 9207067 |
|  | 14764537 |
|  | 15649874 |
|  | 16202150 |
|  | 1592003 |
|  | 2479986 |
|  | 15649872 |
|  | 12032352 |
|  | 15292252 |
|  | 17014841 |
|  | 12888890 |
|  | 17249574 |
|  | 17249573 |
|  | 14519228 |
|  | 12197476 |
|  | 14719080 |
|  | 12093914 |
|  | 15305294 |
|  | 17203162 |
|  | 15665295 |
|  | 14633511 |
|  | 16895970 |
|  | 16470614 |
|  | 11169851 |
|  | 15572520 |
|  | 16026585 |
|  | 17505511 |
|  | 17374694 |
|  | 12007837 |
|  | 16930552 |
|  | 17310988 |
|  | 9012841 |
|  | 12548213 |
|  | 12133520 |
|  | 15161709 |
|  | 15135240 |
|  | 16880208 |
|  | 12133521 |
|  | 12060561 |
|  | 15504909 |
|  | 17543594 |
|  | 15263820 |
|  | 17495954 |
|  | 12651930 |
|  | 15383400 |
|  | 17215071 |
|  | 12719950 |
|  | 15963467 |
|  | 8637916 |
|  | 16517614 |
|  | 15800063 |
|  | 11940521 |
|  | 11907124 |
|  | 14729577 |
|  | 15653207 |
|  | 8119973 |
|  | 14551058 |
|  | 14532971 |
|  | 15540202 |
|  | 15175342 |
|  | 16718361 |
|  | 12477932 |
|  | 2320579 |
|  | 15671530 |
|  | 17119993 |
|  | 10335479 |
|  | 17624309 |
|  | 12654193 |
|  | 12208074 |
|  | 17446232 |
|  | 12654197 |
|  | 16077951 |
|  | 12057925 |
|  | 11784722 |
|  | 15187003 |
|  | 11115828 |
|  | 10027415 |
|  | 15770733 |
|  | 15474072 |
|  | 15583829 |
|  | 9450968 |
|  | 11891801 |
|  | 14871830 |
|  | 15776280 |
|  | 12588999 |
|  | 11169844 |
|  | 15345585 |
|  | 15084519 |
|  | 15958580 |
|  | 15135259 |
|  | 15375582 |
|  | 11867956 |
|  | 16412381 |
|  | 15583822 |
|  | 16385345 |
|  | 8573716 |
|  | 11331305 |
|  | 15800930 |
|  | 15614129 |
|  | 10587525 |
|  | 11815711 |
|  | 14559444 |
|  | 12890722 |
|  | 11875709 |
|  | 15093699 |
|  | 12482858 |
|  | 15949234 |
|  | 12788875 |
|  | 10581076 |
|  | 12871382 |
|  | 17505039 |
|  | 15820807 |
|  | 11809716 |
|  | 11809713 |
|  | 11181640 |
|  | 15329326 |
|  | 12918061 |
|  | 12716911 |
|  | 12474525 |
|  | 12086892 |
|  | 16184401 |
|  | 16538528 |
|  | 17558601 |
|  | 15140761 |
|  | 17227294 |
|  | 12865814 |
|  | 12827055 |
|  | 15583754 |
|  | 12668280 |
|  | 15735682 |
|  | 10428027 |
|  | 16675573 |
|  | 16428272 |
|  | 17293873 |
|  | 12668286 |
|  | 12446667 |
|  | 11741977 |
|  | 16039638 |
|  | 15832056 |
|  | 17015762 |
|  | 11461089 |
|  | 12665660 |
|  | 15273288 |
|  | 17467696 |
|  | 16603731 |
|  | 14729679 |
|  | 8614412 |
|  | 15193536 |
|  | 15785231 |
|  | 11458389 |
|  | 16826426 |
|  | 15621731 |
|  | 12002441 |
|  | 7678805 |
|  | 16054650 |
|  | 15184633 |
|  | 14656883 |
|  | 17016437 |
|  | 14633603 |
|  | 16322068 |
|  | 15670761 |
|  | 15050899 |
|  | 15851615 |
|  | 14593238 |
|  | 12086877 |
|  | 11819817 |
|  | 16456664 |
|  | 15063179 |
|  | 10022836 |
|  | 7647376 |
|  | 12552636 |
|  | 11956584 |
|  | 12845559 |
|  | 15788755 |
|  | 11069235 |
|  | 11943666 |
|  | 17053052 |
|  | 14694016 |
|  | 16269156 |
|  | 16870555 |
|  | 12042276 |
|  | 8602242 |
|  | 8602241 |
|  | 14719060 |
|  | 15175265 |
|  | 15922088 |
|  | 12149254 |
|  | 17336974 |
|  | 17485853 |
|  | 15284224 |
|  | 14513053 |
|  | 15492846 |
|  | 9336848 |
|  | 11163598 |
|  | 11509733 |
|  | 15633213 |
|  | 8607868 |
|  | 12358602 |
|  | 16172168 |
|  | 15156404 |
|  | 17630849 |
|  | 16105646 |
|  | 14729621 |
|  | 17336904 |
|  | 17257729 |
|  | 12391145 |
|  | 16172161 |
|  | 9202027 |
|  | 11861517 |
|  | 12629515 |
|  | 16403913 |
|  | 17379761 |
|  | 16107612 |
|  | 12810642 |
|  | 12415262 |
|  | 12727858 |
|  | 14513045 |
|  | 16569480 |
|  | 15710418 |
|  | 11964307 |
|  | 15892598 |
|  | 15515495 |
|  | 15063730 |
|  | 12195295 |
|  | 9054410 |
|  | 16787408 |
|  | 12591918 |
|  | 14643756 |
|  | 17477366 |
|  | 14735189 |
|  | 14633819 |
|  | 12597245 |
|  | 12571449 |
|  | 16804907 |
|  | 16198352 |
|  | 14983012 |
|  | 17169805 |
|  | 17121786 |
|  | 15073116 |
|  | 15871723 |
|  | 16597608 |
|  | 16496302 |
|  | 17544391 |
|  | 12445161 |
|  | 17068288 |
|  | 17405882 |
|  | 16616080 |
|  | 17583691 |
|  | 12149478 |
|  | 11912219 |
|  | 15457582 |
|  | 12046073 |
|  | 16728464 |
|  | 16773219 |
|  | 16984727 |
|  | 15196703 |
|  | 15140509 |
|  | 16909109 |
|  | 12742981 |
|  | 17591953 |
|  | 16384981 |
|  | 11912213 |
|  | 15782199 |
|  | 17353919 |
|  | 17509611 |
|  | 17549360 |
|  | 13678594 |
|  | 16973968 |
|  | 17392976 |
|  | 17256873 |
|  | 15864753 |
|  | 14718351 |
|  | 10395786 |
|  | 12847526 |
|  | 11978184 |
|  | 12006174 |
|  | 12540967 |
|  | 12912908 |
|  | 15580293 |
|  | 15072995 |
|  | 15765121 |
|  | 16510872 |
|  | 12676581 |
|  | 17275844 |
|  | 15708566 |
|  | 15334691 |
|  | 15201500 |
|  | 10196157 |
|  | 16784838 |
|  | 16611402 |
|  | 11691837 |
|  | 17043385 |
|  | 12168088 |
|  | 14749705 |
|  | 12553038 |
|  | 14659975 |
|  | 15682485 |
|  | 15136139 |
|  | 16877277 |
|  | 15208295 |
|  | 17560563 |
|  | 9351807 |
|  | 12810677 |
|  | 16163059 |
|  | 14674128 |
|  | 14767569 |
|  | 17454603 |
|  | 16786118 |
|  | 12427750 |
|  | 14983237 |
|  | 17600519 |
|  | 17255526 |
|  | 15809749 |
|  | 12089605 |
|  | 15613104 |
|  | 15579459 |
|  | 16044082 |
|  | 17141472 |
|  | 16824955 |
|  | 11427521 |
|  | 16434047 |
|  | 15746194 |
|  | 12427739 |
|  | 12649282 |
|  | 14767538 |
|  | 17240354 |
|  | 15733833 |
|  | 15073150 |
|  | 14676843 |
|  | 17167981 |
|  | 9113979 |
|  | 15149853 |
|  | 16799557 |
|  | 15588162 |
|  | 12615701 |
|  | 17188266 |
|  | 12376368 |
|  | 12607599 |
|  | 15217829 |
|  | 12439912 |
|  | 16962110 |
|  | 16556738 |
|  | 17189520 |
|  | 11304494 |
|  | 7803491 |
|  | 12607595 |
|  | 16627366 |
|  | 15331549 |
|  | 16891392 |
|  | 17035941 |
|  | 1644816 |
|  | 15086936 |
|  | 12067896 |
|  | 15086939 |
|  | 15629128 |
|  | 16164572 |
|  | 15879288 |
|  | 15503825 |
|  | 15073147 |
|  | 16835303 |
|  | 15503824 |
|  | 15613744 |
|  | 16647384 |
|  | 15256791 |
|  | 14676217 |
|  | 15081307 |
|  | 17640598 |
|  | 12187074 |
|  | 12381667 |
|  | 11775025 |
|  | 16825198 |
|  | 11960372 |
|  | 16899768 |
|  | 16734388 |
|  | 11742492 |
|  | 15067085 |
|  | 15726836 |
|  | 12451991 |
|  | 16245301 |
|  | 12174362 |
|  | 14506249 |
|  | 14655184 |
|  | 15219420 |
|  | 14561656 |
|  | 15081313 |
|  | 11744618 |
|  | 12604407 |
|  | 15244100 |
|  | 15940042 |
|  | 12453880 |
|  | 15075216 |
|  | 16732297 |
|  | 16816927 |
|  | 15302276 |
|  | 11744811 |
|  | 15127326 |
|  | 15126502 |
|  | 17434742 |
|  | 15845615 |
|  | 16597689 |
|  | 16107693 |
|  | 11882460 |
|  | 17085437 |
|  | 8621427 |
|  | 15474452 |
|  | 15569052 |
|  | 14693680 |
|  | 15569051 |
|  | 15340161 |
|  | 10878616 |
|  | 16259067 |
|  | 17597658 |
|  | 16682007 |
|  | 16007189 |
|  | 14670179 |
|  | 15112337 |
|  | 16979977 |
|  | 15515423 |
|  | 16226108 |
|  | 15601855 |
|  | 17604022 |
|  | 14500349 |
|  | 15601856 |
|  | 11599061 |
|  | 15494412 |
|  | 16019439 |
|  | 15731179 |
|  | 15143069 |
|  | 16039615 |
|  | 16499871 |
|  | 17103175 |
|  | 16816119 |
|  | 17083617 |
|  | 12372425 |
|  | 17279364 |
|  | 11986954 |
|  | 11934834 |
|  | 14959852 |
|  | 15051508 |
|  | 17109192 |
|  | 16130099 |
|  | 12429966 |
|  | 12763746 |
|  | 16638750 |
|  | 15704107 |
|  | 12048246 |
|  | 12537522 |
|  | 15388333 |
|  | 15339792 |
|  | 15254962 |
|  | 17581219 |
|  | 16036113 |
|  | 16462734 |
|  | 16278385 |
|  | 17570036 |
|  | 12860293 |
|  | 12112468 |
|  | 15674425 |
|  | 15052220 |
|  | 12581744 |
|  | 9634701 |
|  | 17272396 |
|  | 11290292 |
|  | 15020256 |
|  | 16516166 |
|  | 12192060 |
|  | 11549290 |
|  | 14512017 |
|  | 11877311 |
|  | 10803598 |
|  | 15168728 |
|  | 17483438 |
|  | 10685072 |
|  | 12829717 |
|  | 15582599 |
|  | 11217851 |
|  | 11836562 |
|  | 12133473 |
|  | 16583410 |
|  | 12425822 |
|  | 11934855 |
|  | 12901801 |
|  | 11934854 |
|  | 16230500 |
|  | 17611605 |
|  | 15631865 |
|  | 15945504 |
|  | 17513698 |
|  | 15146197 |
|  | 12226475 |
|  | 16788140 |
|  | 15668894 |
|  | 17277913 |
|  | 15879344 |
|  | 16176284 |
|  | 8815891 |
|  | 15374700 |
|  | 16251428 |
|  | 17672245 |
|  | 12858545 |
|  | 14753452 |
|  | 11953313 |
|  | 16613616 |
|  | 16243805 |
|  | 14753438 |
|  | 12163680 |
|  | 12563064 |
|  | 17409493 |
|  | 17671399 |
|  | 12368264 |
|  | 17569125 |
|  | 15374715 |
|  | 12141529 |
|  | 15935515 |
|  | 15202016 |
|  | 12461084 |
|  | 15031673 |
|  | 17289903 |
|  | 12387457 |
|  | 16359995 |
|  | 16423853 |
|  | 2735925 |
|  | 15896327 |
|  | 12235106 |
|  | 11940485 |
|  | 14647449 |
|  | 8631822 |
|  | 16283008 |
|  | 15100155 |
|  | 11705851 |
|  | 12579315 |
|  | 16528367 |
|  | 15150105 |
|  | 15467755 |
|  | 12070272 |
|  | 17264168 |
|  | 12483534 |
|  | 12232762 |
|  | 16818728 |
|  | 12696078 |
|  | 15488701 |
|  | 15374730 |
|  | 16396910 |
|  | 15009103 |
|  | 15920019 |
|  | 16581961 |
|  | 16702519 |
|  | 16782887 |
|  | 11981751 |
|  | 15896317 |
|  | 17584582 |
|  | 12151391 |
|  | 11642726 |
|  | 11831981 |
|  | 17369464 |
|  | 12386279 |
|  | 17530707 |
|  | 16260429 |
|  | 16608813 |
|  | 17614845 |
|  | 12126787 |
|  | 11901189 |
|  | 12456629 |
|  | 17369472 |
|  | 15184386 |
|  | 17457373 |
|  | 16490744 |
|  | 16234975 |
|  | 17324777 |
|  | 15137063 |
|  | 16489641 |
|  | 14742307 |
|  | 12393646 |
|  | 17050862 |
|  | 16115033 |
|  | 9323936 |
|  | 15522212 |
|  | 17065333 |
|  | 17215390 |
|  | 17525262 |
|  | 15040022 |
|  | 15477409 |
|  | 15756443 |
|  | 9806910 |
|  | 11832343 |
|  | 16222693 |
|  | 9166595 |
|  | 17367506 |
|  | 15034080 |
|  | 11920479 |
|  | 11866530 |
|  | 11919510 |
|  | 16202216 |
|  | 11824377 |
|  | 16482425 |
|  | 16752188 |
|  | 16432214 |
|  | 16982461 |
|  | 12413884 |
|  | 15545635 |
|  | 15563592 |
|  | 15793222 |
|  | 15667531 |
|  | 11919509 |
|  | 12779248 |
|  | 12234794 |
|  | 11930687 |
|  | 16027358 |
|  | 14662796 |
|  | 12448348 |
|  | 12039984 |
|  | 16545694 |
|  | 12594002 |
|  | 14715242 |
|  | 16432225 |
|  | 10323857 |
|  | 10705382 |
|  | 12397601 |
|  | 16318581 |
|  | 15090854 |
|  | 8889548 |
|  | 16409998 |
|  | 12207021 |
|  | 16088182 |
|  | 15015550 |
|  | 16940309 |
|  | 11289156 |
|  | 10208743 |
|  | 12881220 |
|  | 17618600 |
|  | 15161630 |
|  | 14663468 |
|  | 12777375 |
|  | 16901919 |
|  | 15453815 |
|  | 15489334 |
|  | 12568850 |
|  | 11705867 |
|  | 11930688 |
|  | 12606532 |
|  | 12466305 |
|  | 16530326 |
|  | 12590639 |
|  | 11804779 |
|  | 17211450 |
|  | 16612574 |
|  | 17553957 |
|  | 14529579 |
|  | 15351965 |
|  | 15610240 |
|  | 16514669 |
|  | 15347673 |
|  | 15191350 |
|  | 14610338 |
|  | 12594815 |
|  | 14585871 |
|  | 16931798 |
|  | 7846076 |
|  | 9922142 |
|  | 15031602 |
|  | 15351862 |
|  | 15777969 |
|  | 11230154 |
|  | 16396768 |
|  | 7490564 |
|  | 16966370 |
|  | 11381259 |
|  | 1550962 |
|  | 17538775 |
|  | 14969389 |
|  | 16920734 |
|  | 12708473 |
|  | 14998491 |
|  | 15687494 |
|  | 17470563 |
|  | 11927538 |
|  | 17418408 |
|  | 16908180 |
|  | 16219308 |
|  | 12844492 |
|  | 17488794 |
|  | 12850503 |
|  | 11472838 |
|  | 12406876 |
|  | 15453394 |
|  | 1711045 |
|  | 12117969 |
|  | 15246970 |
|  | 16171822 |
|  | 15122338 |
|  | 12796773 |
|  | 11016853 |
|  | 12016145 |
|  | 16617130 |
|  | 2610687 |
|  | 16712976 |
|  | 14664702 |
|  | 17654674 |
|  | 16413500 |
|  | 15153787 |
|  | 16740682 |
|  | 16990600 |
|  | 10464055 |
|  | 12736220 |
|  | 16170370 |
|  | 9529250 |
|  | 12102661 |
|  | 15371263 |
|  | 11731620 |
|  | 16806288 |
|  | 10349636 |
|  | 15817666 |
|  | 10716993 |
|  | 12538631 |
|  | 15944181 |
|  | 12900522 |
|  | 16269210 |
|  | 11066091 |
|  | 16672722 |
|  | 14980515 |
|  | 11961303 |
|  | 15458440 |
|  | 16856151 |
|  | 15500293 |
|  | 15118294 |
|  | 15306695 |
|  | 15652753 |
|  | 12181492 |
|  | 15689378 |
|  | 12509426 |
|  | 17013884 |
|  | 12744932 |
|  | 12667326 |
|  | 12711260 |
|  | 12174901 |
|  | 10675351 |
|  | 12551914 |
|  | 17168735 |
|  | 12368217 |
|  | 12556420 |
|  | 15911882 |
|  | 11866538 |
|  | 15161688 |
|  | 12558813 |
|  | 16543500 |
|  | 12482957 |
|  | 15838884 |
|  | 11978667 |
|  | 16038988 |
|  | 15673567 |
|  | 12845630 |
|  | 9916007 |
|  | 11686329 |
|  | 11563986 |
|  | 11124112 |
|  | 17222790 |
|  | 17531158 |
|  | 16761623 |
|  | 14708636 |
|  | 15160911 |
|  | 17392174 |
|  | 14681237 |
|  | 15191555 |
|  | 15501249 |
|  | 16465407 |
|  | 17719546 |
|  | 15492987 |
|  | 16916659 |
|  | 15259010 |
|  | 17487399 |
|  | 16388189 |
|  | 14514674 |
|  | 16793871 |
|  | 12086595 |
|  | 15905202 |
|  | 15882861 |
|  | 14762365 |
|  | 12937129 |
|  | 11972026 |
|  | 11171333 |
|  | 11834731 |
|  | 16320826 |
|  | 16515588 |
|  | 15501243 |
|  | 15826815 |
|  | 10700190 |
|  | 15501241 |
|  | 16972327 |
|  | 11783119 |
|  | 14530359 |
|  | 12400015 |
|  | 15504975 |
|  | 15664512 |
|  | 17616354 |
|  | 15732116 |
|  | 15501235 |
|  | 9012504 |
|  | 16916647 |
|  | 16192634 |
|  | 12482580 |
|  | 17659575 |
|  | 17661998 |
|  | 17023682 |
|  | 15726914 |
|  | 11076861 |
|  | 15956982 |
|  | 16160062 |
|  | 16733790 |
|  | 15685607 |
|  | 15185205 |
|  | 16293653 |
|  | 12883688 |
|  | 11799077 |
|  | 16645174 |
|  | 16339827 |
|  | 14726393 |
|  | 14607712 |
|  | 14512791 |
|  | 12883692 |
|  | 16049374 |
|  | 14656735 |
|  | 10942527 |
|  | 15239258 |
|  | 16164632 |
|  | 17121831 |
|  | 11964077 |
|  | 2584205 |
|  | 12430738 |
|  | 12937141 |
|  | 15120936 |
|  | 12453985 |
|  | 11032698 |
|  | 11937493 |
|  | 17461470 |
|  | 14669345 |
|  | 11806247 |
|  | 17162607 |
|  | 12710853 |
|  | 10575000 |
|  | 15550485 |
|  | 15309707 |
|  | 7768934 |
|  | 12957716 |
|  | 11957147 |
|  | 8702615 |
|  | 14651929 |
|  | 10653008 |
|  | 17353160 |
|  | 16830384 |
|  | 14675094 |
|  | 16684540 |
|  | 15637071 |
|  | 17175233 |
|  | 14517396 |
|  | 16549155 |
|  | 14572781 |
|  | 16648151 |
|  | 16223785 |
|  | 12591230 |
|  | 16164642 |
|  | 16820871 |
|  | 16257644 |
|  | 15015562 |
|  | 16410746 |
|  | 10626738 |
|  | 15016731 |
|  | 17237427 |
|  | 14696965 |
|  | 17490619 |
|  | 14648803 |
|  | 11133233 |
|  | 16299233 |
|  | 16595708 |
|  | 16046504 |
|  | 12539040 |
|  | 15607642 |
|  | 12687277 |
|  | 12767510 |
|  | 16911686 |
|  | 16650815 |
|  | 16355211 |
|  | 14574404 |
|  | 16095053 |
|  | 16650817 |
|  | 17088559 |
|  | 11381139 |
|  | 11839570 |
|  | 12885771 |
|  | 17276402 |
|  | 15516835 |
|  | 16461129 |
|  | 10922068 |
|  | 12038454 |
|  | 12975317 |
|  | 12871881 |
|  | 12852851 |
|  | 16049137 |
|  | 16254334 |
|  | 14635718 |
|  | 14507641 |
|  | 12209725 |
|  | 11999550 |
|  | 15117722 |
|  | 15880052 |
|  | 14672622 |
|  | 15050532 |
|  | 16357200 |
|  | 14981918 |
|  | 14530810 |
|  | 12949011 |
|  | 14635716 |
|  | 15996656 |
|  | 16735996 |
|  | 9878851 |
|  | 15964546 |
|  | 16883594 |
|  | 17453634 |
|  | 12012273 |
|  | 14745002 |
|  | 16569553 |
|  | 14507649 |
|  | 15132990 |
|  | 15477421 |
|  | 12168898 |
|  | 11226286 |
|  | 17471570 |
|  | 12105188 |
|  | 11331753 |
|  | 14507446 |
|  | 15610528 |
|  | 10654595 |
|  | 11839592 |
|  | 15217952 |
|  | 15609122 |
|  | 14747470 |
|  | 17112680 |
|  | 17589348 |
|  | 14747750 |
|  | 12855698 |
|  | 12684660 |
|  | 15880074 |
|  | 12407018 |
|  | 10943840 |
|  | 14704231 |
|  | 11439183 |
|  | 8786112 |
|  | 17175272 |
|  | 17187775 |
|  | 11748975 |
|  | 15788677 |
|  | 8756616 |
|  | 17381847 |
|  | 15613470 |
|  | 15532726 |
|  | 12761846 |
|  | 16707486 |
|  | 12114504 |
|  | 15661856 |
|  | 15701844 |
|  | 12515393 |
|  | 14602779 |
|  | 15217947 |
|  | 15001987 |
|  | 12756808 |
|  | 9153276 |
|  | 11827992 |
|  | 16775835 |
|  | 16835467 |
|  | 12670505 |
|  | 15927453 |
|  | 15754412 |
|  | 17559806 |
|  | 16392034 |
|  | 15475194 |
|  | 12213803 |
|  | 15358155 |
|  | 12213806 |
|  | 12174896 |
|  | 15973775 |
|  | 15616224 |
|  | 15847702 |
|  | 11809675 |
|  | 12700666 |
|  | 15274325 |
|  | 12593846 |
|  | 14667896 |
|  | 12839933 |
|  | 12014632 |
|  | 10027394 |
|  | 15646833 |
|  | 16980969 |
|  | 1567395 |
|  | 15465485 |
|  | 17631682 |
|  | 15951295 |
|  | 16186493 |
|  | 14741347 |
|  | 16086118 |
|  | 8602835 |
|  | 17626901 |
|  | 14988276 |
|  | 15609132 |
|  | 11196464 |
|  | 15916974 |
|  | 8641698 |
|  | 12151344 |
|  | 10813585 |
|  | 15661835 |
|  | 16949073 |
|  | 11956638 |
|  | 15014023 |
|  | 7706320 |
|  | 9665379 |
|  | 15705187 |
|  | 12209585 |
|  | 15180964 |
|  | 12618525 |
|  | 16343055 |
|  | 16482999 |
|  | 16141073 |
|  | 16141072 |
|  | 7759509 |
|  | 11980898 |
|  | 10873597 |
|  | 16084492 |
|  | 10926756 |
|  | 8968091 |
|  | 9861052 |
|  | 11577200 |
|  | 12151311 |
|  | 15614762 |
|  | 17180351 |
|  | 16937522 |
|  | 14550787 |
|  | 15548611 |
|  | 17065555 |
|  | 16601840 |
|  | 17434451 |
|  | 16835828 |
|  | 12493399 |
|  | 17509958 |
|  | 12062186 |
|  | 15886312 |
|  | 17511025 |
|  | 12499259 |
|  | 12646239 |
|  | 17572843 |
|  | 16831171 |
|  | 15347840 |
|  | 7518352 |
|  | 9256442 |
|  | 16394111 |
|  | 16239639 |
|  | 16699073 |
|  | 14607580 |
|  | 17348020 |
|  | 17626736 |
|  | 12053176 |
|  | 17334879 |
|  | 16643910 |
|  | 15456771 |
|  | 10706142 |
|  | 16010973 |
|  | 12168808 |
|  | 14660505 |
|  | 15993383 |
|  | 16839256 |
|  | 16778129 |
|  | 11090062 |
|  | 17478585 |
|  | 7933988 |
|  | 12446788 |
|  | 12481883 |
|  | 12119553 |
|  | 16203926 |
|  | 15001532 |
|  | 16142870 |
|  | 15298982 |
|  | 15562207 |
|  | 16877353 |
|  | 12023772 |
|  | 12893367 |
|  | 15166499 |
|  | 14609725 |
|  | 7683699 |
|  | 12883661 |
|  | 7534783 |
|  | 15768385 |
|  | 11165481 |
|  | 12926153 |
|  | 16411082 |
|  | 12496364 |
|  | 12509854 |
|  | 11032855 |
|  | 11959648 |
|  | 11122379 |
|  | 14764596 |
|  | 16169405 |

---

|  |  |
| --- | --- |
| Tissue | Pancreatic islets |
|  | Tongue |
|  | Kidney |
|  | Pancreas |
|  | Testis interstitial |
|  | Uterus |
|  | Superior cervical ganglion |
|  | Uterus corpus |
|  | Bronchial epithelial cells |
|  | Salivary gland |
|  | Pb-bdca4+dentritic |
|  | Fetal thyroid |
|  | Pb-cd14+monocytes |
|  | Heart |
|  | Cardiac myocytes |
|  | Fetal brain |
|  | Atrioventricular node |
|  | Burkitts-daudi lymphoma |
|  | Adrenal gland |
|  | Appendix |
|  | bone marrow |
|  | Bm-cd105+endothelial |
|  | Bm-cd34+ |
|  | Fetal lung |
|  | Burkitts-raji lymphoma |
|  | Temporal lobe |
|  | Leukemia chronic myelogenous(k562) |
|  | Testis germ cell |
|  | Trigeminal ganglion |
|  | adrenal cortex |
|  | Testis leydig cell |
|  | Skin |
|  | Whole brain |
|  | Pb-cd8+tcells |
|  | Pb-cd56+nkcells |
|  | thyroid |
|  | Placenta |
|  | skeletal muscle |
|  | Olfactory bulb |
|  | Bm-cd33+myeloid |
|  | smooth muscle |
|  | Ciliary ganglion |
|  | Prostate |
|  | Ovary |
|  | Trachea |
|  | Bm-cd71+early erythroid |
|  | Cerebellum |
|  | Lymphnode |
|  | Lung |
|  | Liver |
|  | Colorectal adenocarcinoma |
|  | Lymphoblasts |
|  | Pituitary |
|  | amygdala |
|  | tonsil |
|  | Fetal liver |
|  | Testis seminiferous tubule |

---

|  |  |
| --- | --- |
| Organism | Homo sapiens {Organism urn:agi-taxid:9606} |
|  | Mus musculus {Organism urn:agi-taxid:10090} |
|  | Rattus norvegicus {Organism urn:agi-taxid:10116} |
|  | Homo sapiens |
|  | Mus musculus |
|  | Rattus norvegicus |
|  | Bos taurus |
|  | Danio rerio |

---

|  |  |
| --- | --- |
| Mouse chromosome position | 17 22.79 cM |
|  | 17 24.2 cM |

---

|  |  |
| --- | --- |
| OMIM ID | 192240 |
|  | 603933 |
|  | 125853 |

---

|  |  |
| --- | --- |
| Rat chromosome position | 9q12 |

---

|  |  |
| --- | --- |
| Hugo ID | 12680 |
|  | HGNC:12680 |

---

|  |  |
| --- | --- |
| Human chromosome position | 6p21.1 |
|  | 6p12 |

---

|  |  |
| --- | --- |
| Swiss-Prot Accession | A0A087WUD8 |
|  | P15692 |
|  | A0A087X0B2 |
|  | A0A087WTP4 |
|  | A0A087X1L1 |
|  | A0A024RD33 |
|  | A0A024RD37 |
|  | A0A0Y0IMM4 |
|  | A2A2V4 |
|  | P15692.2 |
|  | A0A1L1SVG2 |
|  | Q00731 |
|  | F6ZE01 |
|  | Q00731.2 |
|  | B5DEK7 |
|  | P16612 |
|  | Q541S7 |
|  | A0A0G2K6Z7 |
|  | P16612.2 |
|  | B5BU86 |
|  | H0Y2S8 |
|  | H0Y407 |
|  | H0Y414 |
|  | H0Y462 |
|  | H0Y8N2 |
|  | H3BLW7 |
|  | O60720 |
|  | O75875 |
|  | Q074Z4 |
|  | Q16889 |
|  | Q5UB46 |
|  | Q6P0P5 |
|  | Q96KJ0 |
|  | Q96L82 |
|  | Q96NW5 |
|  | Q9H1W8 |
|  | Q9H1W9 |
|  | Q9UH58 |
|  | Q9UL23 |
|  | O70123 |
|  | Q66S31 |
|  | Q6GT23 |
|  | Q6WZL9 |
|  | Q541S6 |
|  | Q91ZE1 |
|  | Q9JKX7 |
|  | Q9QXG6 |
|  | Q9QXG7 |
|  | Q59FH5 |
|  | Q6WZM0 |
|  | Q71S09 |
|  | Q96FD9 |
|  | Q9UNS8 |
|  | A0FKR4 |
|  | Q5UD54 |
|  | Q6YLN3 |
|  | Q80UA0 |
|  | O88911 |
|  | Q63672 |
|  | Q64FZ6 |
|  | Q78EG9 |
|  | Q91V21 |
|  | Q91Y66 |
|  | Q91Y68 |
|  | Q91ZE2 |
|  | VEGFA\_HUMAN |
|  | VEGFA\_MOUSE |
|  | VEGFA\_RAT |
|  | B5DEK7\_RAT |
|  | Q541S7\_RAT |

---

|  |  |
| --- | --- |
| PIR ID | A41551 |
|  | A44881 |
|  | B44881 |
|  | A35987 |

---

|  |  |
| --- | --- |
| GenBank ID | NC\_000006 |
|  | NM\_001171623 |
|  | NP\_001165094 |
|  | NM\_001171624 |
|  | NP\_001165095 |
|  | NM\_001204384 |
|  | NP\_001191313 |
|  | NM\_001171625 |
|  | NP\_001165096 |
|  | NM\_001171626 |
|  | NP\_001165097 |
|  | NM\_001317010 |
|  | NP\_001303939 |
|  | NM\_001171629 |
|  | NP\_001165100 |
|  | NM\_001171627 |
|  | NP\_001165098 |
|  | NM\_001171628 |
|  | NP\_001165099 |
|  | NM\_001171630 |
|  | NP\_001165101 |
|  | NM\_001025366 |
|  | NP\_001020537 |
|  | NM\_003376 |
|  | NP\_003367 |
|  | NM\_001204385 |
|  | NP\_001191314 |
|  | NM\_001025367 |
|  | NP\_001020538 |
|  | NM\_001025368 |
|  | NP\_001020539 |
|  | NM\_001033756 |
|  | NP\_001028928 |
|  | NM\_001025369 |
|  | NP\_001020540 |
|  | NM\_001025370 |
|  | NP\_001020541 |
|  | NM\_001171622 |
|  | NP\_001165093 |
|  | NM\_001287044 |
|  | NP\_001273973 |
|  | NG\_008732 |
|  | AF092126 |
|  | AF095785 |
|  | AAD04918 |
|  | AF437895 |
|  | AAL27630 |
|  | AH001553 |
|  | AAA36804 |
|  | AH006909 |
|  | AAC63101 |
|  | AL136131 |
|  | CH471081 |
|  | EAX04227 |
|  | EAX04228 |
|  | EAX04229 |
|  | EAX04230 |
|  | EAX04231 |
|  | EAX04232 |
|  | EAX04233 |
|  | EAX04234 |
|  | EAX04235 |
|  | CQ858312 |
|  | CAH23417 |
|  | CS067226 |
|  | CAI84576 |
|  | CS172363 |
|  | CAJ32889 |
|  | CS172373 |
|  | CAJ33665 |
|  | CS367293 |
|  | CAL32723 |
|  | CS367438 |
|  | CAL33850 |
|  | HC040683 |
|  | CBH30534 |
|  | HC040693 |
|  | CBH30539 |
|  | Y08736 |
|  | AB021221 |
|  | BAA78418 |
|  | AB209485 |
|  | BAD92722 |
|  | AB451322 |
|  | BAG70136 |
|  | AB451451 |
|  | BAG70265 |
|  | AF022375 |
|  | AAC63143 |
|  | AF024710 |
|  | AF062645 |
|  | AAC16730 |
|  | AF091352 |
|  | AAD55345 |
|  | AF214570 |
|  | AAF19659 |
|  | AF323587 |
|  | AAQ14857 |
|  | AF430806 |
|  | AAL27435 |
|  | AF486837 |
|  | AAM03108 |
|  | AJ010438 |
|  | CAA09179 |
|  | AK056914 |
|  | AK125666 |
|  | AY047581 |
|  | AAK95847 |
|  | AY263145 |
|  | AAP86646 |
|  | AY500353 |
|  | AY766116 |
|  | AAV34601 |
|  | BC011177 |
|  | AAH11177 |
|  | BC019867 |
|  | BC058855 |
|  | BC065522 |
|  | AAH65522 |
|  | BF700556 |
|  | BM661679 |
|  | BQ880667 |
|  | BU153227 |
|  | BX442364 |
|  | CN256173 |
|  | CX756573 |
|  | DA237741 |
|  | DQ229900 |
|  | ABB58912 |
|  | HY066915 |
|  | KT581010 |
|  | AMB36732 |
|  | KT581011 |
|  | AMB36733 |
|  | M27281 |
|  | AAA36807 |
|  | M32977 |
|  | AAA35789 |
|  | S85192 |
|  | AAC63102 |
|  | X62568 |
|  | CAA44447 |
|  | P15692 |
|  | NC\_000083 |
|  | NM\_001025257 |
|  | NP\_001020428 |
|  | NM\_001287058 |
|  | NP\_001273987 |
|  | NM\_009505 |
|  | NP\_033531 |
|  | NM\_001287057 |
|  | NP\_001273986 |
|  | NM\_001317041 |
|  | NP\_001303970 |
|  | NM\_001025250 |
|  | NP\_001020421 |
|  | NM\_001287056 |
|  | NP\_001273985 |
|  | NM\_001110268 |
|  | NP\_001103738 |
|  | NM\_001110267 |
|  | NP\_001103737 |
|  | NM\_001110266 |
|  | NP\_001103736 |
|  | AB086118 |
|  | BAC54913 |
|  | AC127690 |
|  | AF317892 |
|  | CH466559 |
|  | EDL23467 |
|  | U41383 |
|  | AK031905 |
|  | AK131850 |
|  | AW913188 |
|  | AY120866 |
|  | AAM55477 |
|  | AY263146 |
|  | AAP86647 |
|  | AY707864 |
|  | AAU11325 |
|  | AY750956 |
|  | AAU95484 |
|  | AY750957 |
|  | AAU95485 |
|  | AY756068 |
|  | AAV32504 |
|  | BC022642 |
|  | BC061468 |
|  | AAH61468 |
|  | BQ554097 |
|  | BQ832724 |
|  | BU920197 |
|  | BY001490 |
|  | CA321456 |
|  | CK626177 |
|  | EF028705 |
|  | ABK27265 |
|  | HQ712122 |
|  | ADY05339 |
|  | M95200 |
|  | AAA40547 |
|  | S37052 |
|  | AAB22252 |
|  | S38083 |
|  | AAB22253 |
|  | S38100 |
|  | AAB22254 |
|  | U50279 |
|  | AAC05442 |
|  | Q00731 |
|  | NC\_005108 |
|  | NR\_105011 |
|  | NM\_031836 |
|  | NP\_114024 |
|  | NM\_001287107 |
|  | NP\_001274036 |
|  | NM\_001287111 |
|  | NP\_001274040 |
|  | NM\_001287112 |
|  | NP\_001274041 |
|  | NM\_001110333 |
|  | NP\_001103803 |
|  | NM\_001287108 |
|  | NP\_001274037 |
|  | NM\_001317043 |
|  | NP\_001303972 |
|  | NM\_001110334 |
|  | NP\_001103804 |
|  | NM\_001287110 |
|  | NP\_001274039 |
|  | NM\_001287113 |
|  | NP\_001274042 |
|  | NM\_001287114 |
|  | NP\_001274043 |
|  | NM\_001110335 |
|  | NP\_001103805 |
|  | NM\_001110336 |
|  | NP\_001103806 |
|  | AC\_000077 |
|  | AAHX01058303 |
|  | AC108326 |
|  | CH473987 |
|  | EDM18768 |
|  | EDM18769 |
|  | EDM18770 |
|  | EDM18771 |
|  | EDM18772 |
|  | EDM18773 |
|  | EDM18774 |
|  | EDM18775 |
|  | EDM18776 |
|  | EDM18777 |
|  | EDM18778 |
|  | EDM18779 |
|  | EDM18780 |
|  | EDM18781 |
|  | EDM18782 |
|  | EDM18783 |
|  | EDM18784 |
|  | EDM18785 |
|  | EDM18786 |
|  | EDM18787 |
|  | EDM18788 |
|  | U22372 |
|  | AF062644 |
|  | AAC16448 |
|  | AF080594 |
|  | AAC36708 |
|  | AF215725 |
|  | AAF19211 |
|  | AF215726 |
|  | AAF19212 |
|  | AF222779 |
|  | AAF25958 |
|  | AF260425 |
|  | AAK49396 |
|  | AF261751 |
|  | AAK49408 |
|  | AY033503 |
|  | AAL07523 |
|  | AY033504 |
|  | AAL07524 |
|  | AY033505 |
|  | AAL07525 |
|  | AY033506 |
|  | AAL07526 |
|  | AY033507 |
|  | AAL07527 |
|  | AY033508 |
|  | AAL07528 |
|  | AY378102 |
|  | AY702972 |
|  | AAU36606 |
|  | BC168708 |
|  | AAI68708 |
|  | BQ203400 |
|  | CB718631 |
|  | CB810370 |
|  | CK357826 |
|  | CK358638 |
|  | CK364938 |
|  | CK602714 |
|  | CO565920 |
|  | CV107730 |
|  | EU040284 |
|  | ABW02818 |
|  | EU040285 |
|  | ABW02819 |
|  | GQ423618 |
|  | ACV52076 |
|  | KT971352 |
|  | APA62567 |
|  | L20913 |
|  | AAA42334 |
|  | M32167 |
|  | AAA41211 |
|  | P16612 |
|  | NC\_018917 |
|  | AMYH02013650 |
|  | M63971 |
|  | M63972 |
|  | M63973 |
|  | M63974 |
|  | M63975 |
|  | M63976 |
|  | M63977 |
|  | M63978 |
|  | BC172307 |
|  | AAI72307 |
|  | EU832814 |
|  | ACE87825 |
|  | GQ129408 |
|  | ACT64542 |
|  | AC\_000039 |
|  | AAHY01132989 |
|  | AAHY01132990 |
|  | AAHY01132991 |
|  | ABBA01050660 |
|  | AC\_000138 |
|  | XM\_005249363 |
|  | XP\_005249420 |
|  | AABR06058825 |
|  | CAC19516 |
|  | CAC19515 |
|  | CAC19512 |
|  | CAC19513 |
|  | CAM28207 |
|  | NT\_007592 |
|  | NW\_001838981 |
|  | NW\_923073 |
|  | AC\_000049 |
|  | S85224 |
|  | CR592173 |
|  | CR614384 |
|  | Q59FH5 |
|  | Q6WZM0 |
|  | Q71S09 |
|  | Q96FD9 |
|  | Q9UNS8 |
|  | NT\_039649 |
|  | NW\_001030618 |
|  | A0FKR4 |
|  | Q5UD54 |
|  | Q6YLN3 |
|  | Q80UA0 |
|  | NW\_047813 |
|  | NW\_001084880 |
|  | O88911 |
|  | Q541S7 |
|  | Q63672 |
|  | Q64FZ6 |
|  | Q78EG9 |
|  | Q91V21 |
|  | Q91Y66 |
|  | Q91Y68 |
|  | Q91ZE2 |
|  | 108742042 |
|  | 26352764 |
|  | 32699992 |
|  | 32699990 |
|  | 3719221 |
|  | DQ150576 |
|  | AW045851 |
|  | AAI39620 |
|  | 53850882 |
|  | 340215 |
|  | 53850884 |
|  | 81903235 |
|  | 81903237 |
|  | 81903236 |
|  | 119624636 |
|  | 119624637 |
|  | 119624634 |
|  | 119624635 |
|  | 119624638 |
|  | 62088550 |
|  | 119624639 |
|  | NT\_086693 |
|  | 119624632 |
|  | 119624633 |
|  | NW\_001511339 |
|  | 56207996 |
|  | 3647281 |
|  | 68300769 |
|  | 74749493 |
|  | 3712669 |
|  | 81867318 |
|  | AK194109 |
|  | 37545172 |
|  | BC139619 |
|  | 181971 |
|  | Q5RHW5 |
|  | 119624640 |
|  | 3712671 |
|  | BAE43236 |
|  | 74712795 |
|  | 81871539 |
|  | 81871538 |
|  | 81867478 |
|  | 340301 |
|  | Q8C2W6 |
|  | NP\_444482 |
|  | 137819 |
|  | 204288 |
|  | 13435566 |
|  | 148701219 |
|  | 148701218 |
|  | 81903015 |
|  | 6708500 |
|  | BG073936 |
|  | AAA30805 |
|  | AAA30804 |
|  | NW\_001494158 |
|  | 81886901 |
|  | 19909065 |
|  | 116794760 |
|  | M31836 |
|  | 148701220 |
|  | 148701221 |
|  | 81174728 |
|  | BC004660 |
|  | 5901561 |
|  | 54401695 |
|  | 38181775 |
|  | 123915392 |
|  | 37659 |
|  | Q99MS7 |
|  | 11989985 |
|  | 11989982 |
|  | 11989983 |
|  | 3643123 |
|  | Q99MS6 |
|  | Q99MS5 |
|  | CAC19514 |
|  | Q541S6 |
|  | NT\_082868 |
|  | 54299287 |
|  | 347168 |
|  | 81887482 |
|  | 81887483 |
|  | 149069339 |
|  | 149069338 |
|  | 149069337 |
|  | 149069336 |
|  | NT\_039655 |
|  | 3133495 |
|  | 81895034 |
|  | 149069335 |
|  | 27544279 |
|  | 149069334 |
|  | AI606078 |
|  | 149069333 |
|  | 149069332 |
|  | 149069331 |
|  | Q1ECU5 |
|  | NC\_007115 |
|  | 149069330 |
|  | AK013080 |
|  | AK089917 |
|  | 149069329 |
|  | 149069328 |
|  | 121944495 |
|  | 149069327 |
|  | NP\_776641 |
|  | NC\_007127 |
|  | AAK32109 |
|  | 81890482 |
|  | 82196427 |
|  | 74213693 |
|  | 81876237 |
|  | 4154291 |
|  | NM\_174216 |
|  | 17380502 |
|  | 74741195 |
|  | AAK32112 |
|  | AAK32110 |
|  | XM\_001334409 |
|  | AAK32111 |
|  | DQ026828 |
|  | 52301743 |
|  | AK169974 |
|  | 123797854 |
|  | NW\_000140 |
|  | 81889721 |
|  | 16612070 |
|  | AK087817 |
|  | 148691520 |
|  | M33750 |
|  | AC\_000041 |
|  | NW\_000130 |
|  | AAA30502 |
|  | 163007 |
|  | 163811 |
|  | XM\_001334310 |
|  | 13925663 |
|  | 13925623 |
|  | AAH04660 |
|  | Q99J97 |
|  | 163809 |
|  | NC\_000085 |
|  | NC\_007324 |
|  | BX324164 |
|  | 123241442 |
|  | 81893496 |
|  | 15822721 |
|  | 123906799 |
|  | 15822725 |
|  | 15822723 |
|  | BB099233 |
|  | AF305087 |
|  | AF305088 |
|  | AF305089 |
|  | NM\_053252 |
|  | 15822717 |
|  | 15822719 |
|  | 15822715 |
|  | 33340525 |
|  | 202351 |
|  | 17380528 |
|  | AF305090 |
|  | NM\_001044855 |
|  | BC117665 |
|  | NW\_001513217 |
|  | M32976 |
|  | 110611814 |
|  | Q3V443 |
|  | 75858341 |
|  | EDL33166 |
|  | CH466612 |
|  | EDL33167 |
|  | EDL33168 |
|  | EDL33165 |
|  | 110611802 |
|  | AAI17666 |
|  | 6631029 |
|  | AA959550 |
|  | XP\_001334346 |
|  | 15422109 |
|  | ABA28744 |
|  | 145337885 |
|  | 2951983 |
|  | 1718156 |
|  | 16660686 |
|  | Q1ANK8 |
|  | AC134563 |
|  | 249861 |
|  | 249857 |
|  | 249859 |
|  | 4996351 |
|  | BAC40012 |
|  | CAI19965 |
|  | 13604145 |
|  | 6606566 |
|  | 13604143 |
|  | 6606564 |
|  | P15691 |
|  | 13604149 |
|  | 13604147 |
|  | 51860167 |
|  | NW\_001030639 |
|  | NP\_001038320 |
|  | CAI20661 |
|  | 123781165 |
|  | 81865004 |
|  | 3139081 |
|  | 149069347 |
|  | 149069346 |
|  | AAY89335 |
|  | 149069345 |
|  | XP\_001334445 |
|  | 149069344 |
|  | 149069343 |
|  | 149069342 |
|  | 149069341 |
|  | 149069340 |
|  | AK217724 |
|  | 81872143 |
|  | 74721192 |
|  | 113931650 |
|  | 125838831 |
|  | 125838833 |
|  | 113931651 |
|  | 126007509 |
|  | 126006804 |
|  | 558154 |
|  | Dr.79865 |
|  | ZDB-GENE-041210-111 |
|  | dre:558154 |
|  | 797569 |
|  | AF016244 |
|  | AF059661 |
|  | NM\_131408 |
|  | dre:797569 |

---

|  |  |
| --- | --- |
| Swiss-Prot ID | VEGFA\_HUMAN |
|  | VEGFA\_MOUSE |
|  | VEGFA\_RAT |
|  | Q541S7\_RAT |
|  | B5DEK7\_RAT |

---

|  |  |
| --- | --- |
| Cell Localization | Secreted |
|  | Cell membrane |
|  | Extracellular |

---

|  |  |
| --- | --- |
| Ensembl ID | ENSG00000112715 |
|  | ENSP00000361125.4 |
|  | ENST00000372055.8 |
|  | ENSP00000389864.3 |
|  | ENST00000413642.7 |
|  | ENSP00000361137.3 |
|  | ENST00000372067.7 |
|  | ENSP00000388663.2 |
|  | ENST00000417285.6 |
|  | ENSP00000361134.4 |
|  | ENST00000372064.8 |
|  | ENSP00000421561.1 |
|  | ENST00000482630.6 |
|  | ENSP00000317598.6 |
|  | ENST00000324450.10 |
|  | ENSP00000430479.1 |
|  | ENST00000523873.5 |
|  | ENSP00000428321.1 |
|  | ENST00000520948.5 |
|  | ENSP00000430829.1 |
|  | ENST00000518689.5 |
|  | ENSP00000429643.1 |
|  | ENST00000523950.5 |
|  | ENSP00000429008.1 |
|  | ENST00000523125.5 |
|  | ENSP00000361148.4 |
|  | ENST00000372077.8 |
|  | ENSP00000430002.1 |
|  | ENST00000518824.5 |
|  | ENSP00000409911.2 |
|  | ENST00000457104.6 |
|  | ENSP00000430594.1 |
|  | ENST00000519767.5 |
|  | ENSP00000230480.6 |
|  | ENST00000230480.10 |
|  | ENSP00000388465.2 |
|  | ENST00000425836.6 |
|  | ENSMUSG00000023951 |
|  | ENSMUSP00000115883.2 |
|  | ENSMUST00000142351.8 |
|  | ENSMUSP00000131901.2 |
|  | ENSMUST00000167860.8 |
|  | ENSMUSP00000109148.1 |
|  | ENSMUST00000113520.7 |
|  | ENSMUSP00000024747.7 |
|  | ENSMUST00000024747.13 |
|  | ENSMUSP00000071575.5 |
|  | ENSMUST00000071648.11 |
|  | ENSRNOG00000019598 |
|  | ENSRNOP00000026559.3 |
|  | ENSRNOT00000026559.5 |
|  | ENSRNOP00000050127.2 |
|  | ENSRNOT00000044163.4 |
|  | ENSRNOP00000047820.3 |
|  | ENSRNOT00000042790.3 |
|  | ENSRNOP00000074002.1 |
|  | ENSRNOT00000083811.1 |
|  | ENSRNOP00000026637.4 |
|  | ENSRNOT00000026637.7 |
|  | ENSP00000478570 |
|  | ENST00000611736 |
|  | ENSP00000389864 |
|  | ENST00000413642 |
|  | ENSP00000483241 |
|  | ENST00000621747 |
|  | ENSP00000388663 |
|  | ENST00000417285 |
|  | ENSP00000478034 |
|  | ENST00000615393 |
|  | ENSP00000421561 |
|  | ENST00000482630 |
|  | ENSP00000484284 |
|  | ENST00000617771 |
|  | ENSP00000430479 |
|  | ENST00000523873 |
|  | ENSP00000428321 |
|  | ENST00000520948 |
|  | ENSP00000430829 |
|  | ENST00000518689 |
|  | ENSP00000429643 |
|  | ENST00000523950 |
|  | ENSP00000429008 |
|  | ENST00000523125 |
|  | ENSP00000361148 |
|  | ENST00000372077 |
|  | ENSP00000430002 |
|  | ENST00000518824 |
|  | ENSP00000409911 |
|  | ENST00000457104 |
|  | ENSP00000430594 |
|  | ENST00000519767 |
|  | ENSP00000230480 |
|  | ENST00000230480 |
|  | ENSP00000388465 |
|  | ENST00000425836 |
|  | ENSMUSP00000115883 |
|  | ENSMUST00000142351 |
|  | ENSMUSP00000131901 |
|  | ENSMUST00000167860 |
|  | ENSMUSP00000109148 |
|  | ENSMUST00000113520 |
|  | ENSMUSP00000024747 |
|  | ENSMUST00000024747 |
|  | ENSMUSP00000071575 |
|  | ENSMUST00000071648 |
|  | ENSRNOP00000026559 |
|  | ENSRNOT00000026559 |
|  | ENSRNOP00000050127 |
|  | ENSRNOT00000044163 |
|  | ENSRNOP00000047820 |
|  | ENSRNOT00000042790 |
|  | ENSRNOP00000074002 |
|  | ENSRNOT00000083811 |
|  | ENSRNOP00000026637 |
|  | ENSRNOT00000026637 |
|  | ENST00000372067 |
|  | ENSP00000361137 |
|  | ENSP00000361134 |
|  | ENST00000372064 |
|  | ENST00000324450 |
|  | ENSP00000361125 |
|  | ENST00000372055 |
|  | ENSP00000317598 |

---

|  |  |
| --- | --- |
| MGI ID | MGI:103178 |
|  | 103178 |
|  | 3612340 |

---

|  |  |
| --- | --- |
| RGD ID | 619991 |

---

|  |  |
| --- | --- |
| Unigene ID | Rn.1923 |
|  | Mm.282184 |
|  | Hs.73793 |
|  | Mm.477156 |
|  | Dr.79865 |
|  | Mm.210447 |
|  | Mm.470969 |
|  | Bt.4138 |

---

|  |  |
| --- | --- |
| Homologene ID | 2534 |

---

|  |  |
| --- | --- |
| Shape | Rhomb |

---

|  |  |
| --- | --- |
| IPI ID | IPI00402915 |
|  | IPI00656297 |
|  | IPI00231977 |
|  | IPI00607893 |
|  | IPI00012567 |
|  | IPI00954880 |
|  | IPI00955048 |
|  | IPI00954971 |
|  | IPI00852889 |
|  | IPI00955379 |
|  | IPI00940477 |
|  | IPI00214604 |
|  | IPI00829762 |
|  | IPI00327982 |
|  | IPI00216555 |
|  | IPI00954917 |
|  | IPI00216553 |
|  | IPI00475372 |
|  | IPI00209785 |
|  | IPI00989633 |
|  | IPI00954943 |
|  | IPI00374772 |
|  | IPI00989242 |
|  | IPI00915448 |
|  | IPI00872936 |
|  | IPI00555908 |
|  | IPI00793077 |
|  | IPI00436015 |
|  | IPI00216554 |
|  | IPI00064652 |
|  | IPI00607783 |
|  | IPI00467967 |
|  | IPI00223442 |
|  | IPI00877296 |
|  | IPI00877306 |
|  | IPI00877273 |
|  | IPI00831263 |
|  | IPI00830961 |
|  | IPI00214186 |
|  | IPI00199066 |
|  | IPI00877356 |

---

|  |  |
| --- | --- |
| Microarray ID | 212171\_x\_at |
|  | 103520\_at |
|  | 210512\_s\_at |
|  | 1416804\_at |
|  | 1448467\_a\_at |
|  | 1451959\_a\_at |

---

|  |  |
| --- | --- |
| Homo sapiens Chromosome position | 6p12 |

---

|  |  |
| --- | --- |
| Ensembl Transcript ID | ENST00000372055 |
|  | ENST00000372064 |
|  | ENST00000372067 |
|  | ENST00000531781 |
|  | ENST00000372077 |
|  | ENST00000520948 |
|  | ENST00000523950 |
|  | ENST00000523873 |
|  | ENST00000457104 |
|  | ENST00000518689 |
|  | ENST00000425836 |
|  | ENST00000518824 |
|  | ENST00000324450 |
|  | ENST00000523125 |
|  | ENST00000531362 |
|  | ENST00000413642 |
|  | ENST00000532514 |
|  | ENST00000482630 |
|  | ENST00000530947 |
|  | ENST00000417285 |

---

|  |  |
| --- | --- |
| FunctionalClass | heparin binding |
|  | protein binding |
|  | extracellular matrix binding |
|  | protein homodimerization |
|  | vascular endothelial growth factor receptor binding |
|  | growth factor |

---

|  |  |
| --- | --- |
| Bos taurus Chromosome position | 23 |

---

|  |  |
| --- | --- |
| Mus musculus Chromosome position | 17 24.2 cM |
|  | 19 A |

---

|  |  |
| --- | --- |
| KEGG pathway | Renal cell carcinoma |
|  | Focal adhesion |
|  | Pancreatic cancer |
|  | mTOR signaling pathway |
|  | VEGF signaling pathway |

---

|  |  |
| --- | --- |
| Rattus norvegicus Chromosome position | 9q12 |

---

|  |  |
| --- | --- |
| Ensembl Gene ID | ENSG00000112715 |

---

|  |  |
| --- | --- |
| LocatorString | pmftq:oncomed |
|  | pmquery:invasive+breast+cancer |
|  | digtext:C:\Users\Ariadne\Customers\Elan\Elan weekly report from literature condensed.txt |
|  | gscquery:curcumin+prostate+cancer |

---
